# Physiological and Molecular Approaches for Developing Thermotolerance in Vegetable Crops: A Growth, Yield and Sustenance Perspective

**DOI:** 10.3389/fpls.2022.878498

**Published:** 2022-06-28

**Authors:** Shikha Chaudhary, Poonam Devi, Bindumadhava HanumanthaRao, Uday Chand Jha, Kamal Dev Sharma, P. V. Vara Prasad, Shiv Kumar, Kadambot H. M. Siddique, Harsh Nayyar

**Affiliations:** ^1^Department of Botany, Panjab University, Chandigarh, India; ^2^World Vegetable Center, International Crops Research Institute for the Semi-Arid Tropics (ICRISAT), Greater Hyderabad, Hyderabad, India; ^3^Marri Channa Reddy Foundation (MCRF), Hyderabad, India; ^4^Crop Improvement Division, Indian Institute of Pulses Research, Kanpur, India; ^5^Department of Agricultural Biotechnology, Chaudhary Sarwan Kumar Himachal Pradesh Agricultural University, Palampur, India; ^6^Department of Agronomy, Kansas State University, Manhattan, KS, United States; ^7^International Center for Agriculture Research in the Dry Areas (ICARDA), Rabat, Morocco; ^8^The University of Western Australia Institute of Agriculture, The University of Western Australia, Perth, WA, Australia

**Keywords:** high temperature, vegetables, heat, environment, climate change

## Abstract

Vegetables are a distinct collection of plant-based foods that vary in nutritional diversity and form an important part of the healthy diet of the human being. Besides providing basic nutrition, they have great potential for boosting human health. The balanced consumption of vegetables is highly recommended for supplementing the human body with better nutrition density, dietary fiber, minerals, vitamins, and bioactive compounds. However, the production and quality of fresh vegetables are influenced directly or indirectly by exposure to high temperatures or heat stress (HS). A decline in quality traits and harvestable yield are the most common effects of HS among vegetable crops. Heat-induced morphological damage, such as poor vegetative growth, leaf tip burning, and rib discoloration in leafy vegetables and sunburn, decreased fruit size, fruit/pod abortion, and unfilled fruit/pods in beans, are common, often rendering vegetable cultivation unprofitable. Further studies to trace down the possible physiological and biochemical effects associated with crop failure reveal that the key factors include membrane damage, photosynthetic inhibition, oxidative stress, and damage to reproductive tissues, which may be the key factors governing heat-induced crop failure. The reproductive stage of plants has extensively been studied for HS-induced abnormalities. Plant reproduction is more sensitive to HS than the vegetative stages, and affects various reproductive processes like pollen germination, pollen load, pollen tube growth, stigma receptivity, ovule fertility and, seed filling, resulting in poorer yields. Hence, sound and robust adaptation and mitigation strategies are needed to overcome the adverse impacts of HS at the morphological, physiological, and biochemical levels to ensure the productivity and quality of vegetable crops. Physiological traits such as the stay-green trait, canopy temperature depression, cell membrane thermostability, chlorophyll fluorescence, relative water content, increased reproductive fertility, fruit numbers, and fruit size are important for developing better yielding heat-tolerant varieties/cultivars. Moreover, various molecular approaches such as omics, molecular breeding, and transgenics, have been proved to be useful in enhancing/incorporating tolerance and can be potential tools for developing heat-tolerant varieties/cultivars. Further, these approaches will provide insights into the physiological and molecular mechanisms that govern thermotolerance and pave the way for engineering “designer” vegetable crops for better health and nutritional security. Besides these approaches, agronomic methods are also important for adaptation, escape and mitigation of HS protect and improve yields.

## Introduction

Vegetables are parts of plants cultivated worldwide for consumption as flowers (e.g., cauliflower, broccoli), fruits (e.g., okra, tomato, cucumber, capsicum), leaves (e.g., spinach, lettuce, brassica, cabbage), tubers (e.g., potato, sweet potato), pods and seeds (e.g., common bean, chickpea, broad bean, mungbean, peas) (Peet and Wolfe, 2000). Vegetables contain secondary metabolites with bioactive properties, including carotenoids (e.g., carrots, pepper, tomato, spinach), polyphenols (e.g., tomato, cabbage), glucosinolates (e.g., brassica), saponins (e.g., beans, pea), and terpenes (e.g., carrots, tomato) (Crozier et al., 2006). These bioactive compounds are metabolic intermediates of primary metabolic processes, which are not essential for plant growth but are used in plant defense responses and plant-insect interactions and can stimulate human health. Clearly, vegetables are an important part of the human diet as they replenish our body with various nutrients, including vitamins, dietary minerals, fibers, proteins, antioxidants, carbohydrates, small amounts of fat, and phytochemicals with anticarcinogenic, antiviral, antifungal, and antibacterial properties (Osagie and Eka, 1998; Teng et al., 2021). While not a major energy source, vegetables nourish our bodies with much-needed minerals and vitamins. According to Food and Agriculture Organization (FAO) statistics, vegetables are the source of dietary requirements about 60% of vitamin A and 90% of vitamin C (Gruda, 2005). Vegetables can earn extra income for farmers as they are seasonal plants with higher yields per hectare than staple crops (Abewoy, 2018). The market value of vegetables is assessed by their quality; FAO and WHO provide many quality attributes for grading vegetables, e.g., color, size, shape, texture, aroma, shelf life, and storability (Gruda, 2005). Vegetables are categorized into two groups according to their growing season; warm-season vegetables include capsicum, common bean, cucumber, cowpea, okra, tomato, and mungbean (Peet and Wolfe, 2000), while cool-season vegetables include brassica, broad bean, broccoli, cabbage, cauliflower, lettuce, radish, spinach, soybean, pea, and potato (Peet and Wolfe, 2000) (Table 1).

**Table 1:** Threshold temperature for some vegetable crops at different stages of plant development.

**Crop**	**Family**	**Threshold** **temperature (**°**C)**	**Response**	**References**
***Cool season vegetables***				
**Vegetative stage**				
**Broccoli** (*Brassica oleracea* var. *italica*)	Brassicaceae	30°C	Reduced growth and development	Hatfield and Prueger, 2015
**Cabbage** (*Brassica oleracea* var. *capitata*)	Brassicaceae	30°C	Reduced growth and development	Warland et al., 2006
**Cauliflower** (*Brassica oleracea* var. *botrytis*)	Brassicaceae	25°C	Reduced leaf growth	Lin et al., 2015
**Reproductive stage**				
**Brassica** (*Brassica napus*)	Brassicaceae	29°C	Reduction in flower number	Morrison and Stewart, 2002
**Broad bean** (*Viciafaba*)	Fabaceae	30/22°C	Accelerate Floral development	Bishop et al., 2016
**Broccoli** (*Brassica oleracea* var. *italica*)	Brassicaceae	35°C	Arrest of inflorescence development	Björkman and Pearson, 1998
**Seed filling/maturity stage**				
**Chickpea** (*Cicer arietinum* L.)	Fabaceae	30°C	Reduced yield	Summerfield and Wien, 1980
**Lettuce** (*Lactuca sativa*)	Asteraceae	24°C	Reduced yield	Jenni, 2005
**Pea** (*Pisum sativum*)	Fabaceae	25.6°C	Reduced yield	Pumphrey and Ramig, 1990
**Potato** (*Solanum tuberosum*)	Solanaceae	30/20°C	Reduced yield	Hancock et al., 2014
***Warm season vegetables***				
**Vegetative stage**				
**Cucumber** (*Cucumis sativus*)	Cucurbitaceae	38°C	Impede growth and development	Yu et al., 2022
**Okra** (*Abelmoschus esculentus*)	Malvaceae	35°C	Decreased leaf size	Hayamanesh, 2018
**Reproductive stage**				
**Capsicum** (*Capsicum annuum* L.)	Solanaceae	33°C	Inhibition of fertilization or early fruit development	Erickson and Markhart, 2002
**Common bean** (*Phaseolus vulgaris*)	Fabaceae	34/24°C	Reduced pollen viability	Boote et al., 2005
**Soybean** (*Glycine max*)	Fabaceae	26/20°C	Delay flowering and distort pod development	Nahar et al., 2016
**Tomato** (*Lycopersicon esculentum*)	Solanaceae	32/26°C	Abnormalities in male and female reproductive tissues	Peet et al., 1998
**Seed filling/maturity stage**				
**Cowpea** (*Vigna unguiculata*)	Fabaceae	36/27°C	Reduced yield	Craufurd et al., 1998
**Okra** (*Abelmoschsusesculentus*)	Malvaceae	35°C	Reduced yield	Hayamanesh, 2018

Like other crops, vegetables are also affected by environmental changes that can render vegetable cultivation unprofitable. Abiotic stresses, mainly the high temperature (heat stress. HS), severely limit crop quantity, quality, nutritional status, and production (Boote et al., 2005; Aleem et al., 2021). High temperatures affect the overall growth and development of vegetable crops by altering morphology, physiology, and enzymatic activities. Heat stress (HS) accelerates phenology, shortening the vegetative and reproductive stages. HS reduces vegetable quality, such as changing the color and texture of fruits (e.g., cucumber, pepper, and tomato) (Zipelevish et al., 2000). In general, HS affects morphological, physiological, and biochemical processes of the plant by hampering photosynthetic activity, source-sink relationship, and altered enzymatic activities (Bita and Gerats, 2013; Janni et al., 2020). The quality of vegetables is also impacted by HS, through a change in color and texture of fruit (e.g., cucumber, pepper, and tomato) (Zipelevish et al., 2000). HS also affects the nutritional status of vegetables; for instance, reducing lycopene in tomato (Gross, 1991) and β-carotene in spinach and lettuce (Oyama et al., 1999) and increasing nitrate levels to harmful levels for human consumption.

Due to climate change, in most regions of the world, rising temperatures will decrease quantity and quality of vegetables crops. Studies of Waithaka et al. (2013) suggested that changes in the climate (increased temperatures) will also provide avenues to grow crops in areas where they could not be grown previously. Climate change scenarios further suggest that development of crop and cultivar choice—especially for water-limited or high-temperature areas—will be an important strategy to have adequate yields under changing climate (Thomas et al., 2007). Hence, targeted studies are needed to assess the impact of high-temperature stress on the growth, yield, and quality (taste, flavor, color, nutritional content) of vegetable crops, with suitable agronomic strategies, developed to create heat-tolerant cultivars or mitigate HS.

## Heat Stress and Vegetables

High temperatures adversely impact plant growth and development (Hasanuzzaman et al., 2013). The constantly rising average surface temperature due to global warming is stressful for all plant growth and development phases, limiting metabolism and productivity, particularly in tropical and subtropical countries (Li et al., 2018). According to the newly released sixth assessment report of IPCC (2021), temperature during the twenty-first century is likely to increase by 1.5°C of warming within just the next two decades, and by 4.5°C, depending on the rate of greenhouse gas emissions. As plants are sedentary organisms, they acclimate to HS by using avoidance mechanisms or programmed cell death (Mittler et al., 2012; Singh, 2013; Zhang T. et al., 2020). Each vegetable crop has temperature threshold for its growth and development; HS will occur beyond the upper threshold for temperature (Wahid et al., 2007; Prasad et al., 2008, 2017). HS impedes photosynthesis through reduced carbon assimilation, ATP reduction, and oxidative damage to chloroplasts, with simultaneous reductions in dry matter accumulation and yield (Sharkey, 2005; Farooq et al., 2011). HS adversely affects vegetative and reproductive plant parts (Bita and Gerats, 2013); thus, the impact of HS varies depending on the developmental stage and crop species (Prasad et al., 2017; Li et al., 2018) (Table 2).

**Table 2:** Noticeable symptoms of heat stress in some vegetable crops.

**Crop species**	**Symptoms**	**References**
**Cabbage** (*Brassica oleracea* var. *capitata*)	Loosening or bolting of heads, smaller and tighter heads, rough leaf texture	Chang et al., 2016
**Capsicum** (*Capsicum annuum*)	Sun scald, yellowing and wilting	Moretti et al., 2010
**Cauliflower** (*Brassica oleracea* var. *botrytis*)	Leafy and uneven heads, puffy buds, yellow eyes and leaves, narrow leaves and hollow stems	Lin et al., 2015
**Common bean** (*Phaseolus vulgaris*)	High fiber in pods, brown and reddish spots in pods	Moretti et al., 2010
**Lettuce** (*Lactuca sativa*)	Tip burn, bolting, loose puffy heads, decreases β-carotene content	Han et al., 2013
**Potato** (*Solanum tuberosum*)	Secondary growth and heat sprouting	Hancock et al., 2014
**Spinach** (*Spinacia oleracea*)	Reduced leaf area and shoots dry weight, reduces β-carotene content	Chitwood et al., 2016
**Tomato** (*Lycopersicon* *esculentum*)	Fruit cracking, sunscald, hampered lycopene synthesis, blossom end rot, internal white tissue, blotchy ripening,	Moretti et al., 2010

## Impact on Vegetative Growth

**Figure 1:**
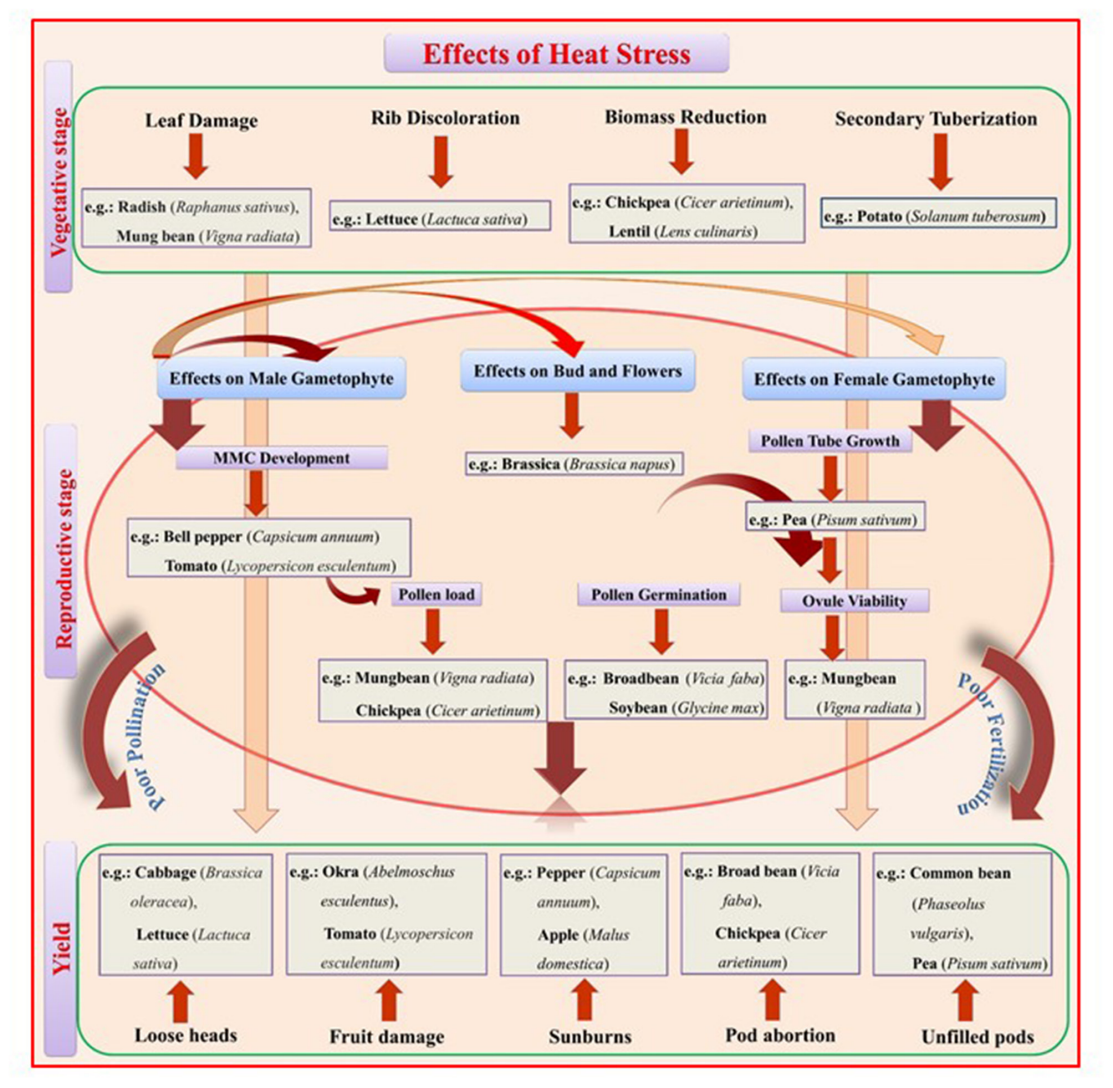
A schematic representation of the effects of heat stress (HS) on vegetative and reproductive growth stages that reduce yield. Heat stress at the vegetative stage promotes leaf damage, rib discoloration in leafy vegetables, biomass reduction in food legumes, and secondary tuberization in potato. Heat stress at the reproductive stage negatively affects the overall route from Microspore Mother Cell (MMC) development to fruit setting/seed filling through pollination and fertilization. The male gametophyte is more prone to heat stress, leading to poor pollen germination, pollen load, and pollen tube growth inside the style and inability to fertilize the ovule at the required rate.

Moderate high temperatures stimulate early vegetative growth and accelerate physiological maturity (Nahar et al., 2015). During seed germination, HS reduces germination percentage and seedling emergence, reduces radical and plumule growth in germinated seedlings, and causes abnormal seedlings and poor seedling vigor (Hasanuzzaman et al., 2013). At later stages of vegetative growth, HS reduces plant height, leaf area, and leaf, stem, pod, root, and total biomass (Kumar et al., 2013). Leafy vegetables require proper growth and development of vegetative parts for realizing only the yield but also the quality. In 45-day-old cabbage plants exposed to 40°C for 6, 12, 24, 48, or 72 h, HS caused loosening or bolting of heads, smaller and tighter heads, and rougher leaf texture (Chang et al., 2016). Likewise, in 30-day-old cauliflower plants exposed to 40°C for 6, 12, 24, 48, 72, or 96 h, HS caused uneven heads, puffy buds, yellow eyes, narrow leaves, reduced leaf growth, and reduced petiole-to-blade ratio (Lin et al., 2015). HS (34.5°C) further delayed the curd induction stage and decreased the chlorophyll content in cauliflower plants; effects were more distinct in heat susceptible genotypes where they were unable to develop curd at high temperature and continued their vegetative growth until temperature fall below 30°C (Aleem et al., 2021). Exposing 4- to 5-leaved lettuce seedlings to 42/37°C for 3 days reduced seedling germination and caused tip burn, rib discoloration, and bolting (Jenni and Yan, 2009; Han et al., 2013). In spinach exposed to 35°C for 21 days, HS decreased seed germination (Chitwood et al., 2016). In potato, high temperature (30–40°C) inhibited tuber development and blocked the tuberization signal (Reynolds and Ewing, 1989). Potato plants exposed to 30/20°C (day/night) for 1 week had reduced yields by 16% compared to plants grown at 22/16°C due to decreased carbon transport to the sink organ (Hancock et al., 2014). Further, reduced yield has been reported in 50 potato cultivars when exposed to heat stressed conditions (35/28°C) than control conditions (22/18°C) (Zhang G. et al., 2020). Likewise, in 6–7-leaved radish seedlings exposed to 40°C for 12 and 24 h, HS affected fleshy taproot growth and development, reducing quality and yield (Zhang et al., 2013) (Figure 1).

## Impact on Reproductive Growth

**Figure 2:**
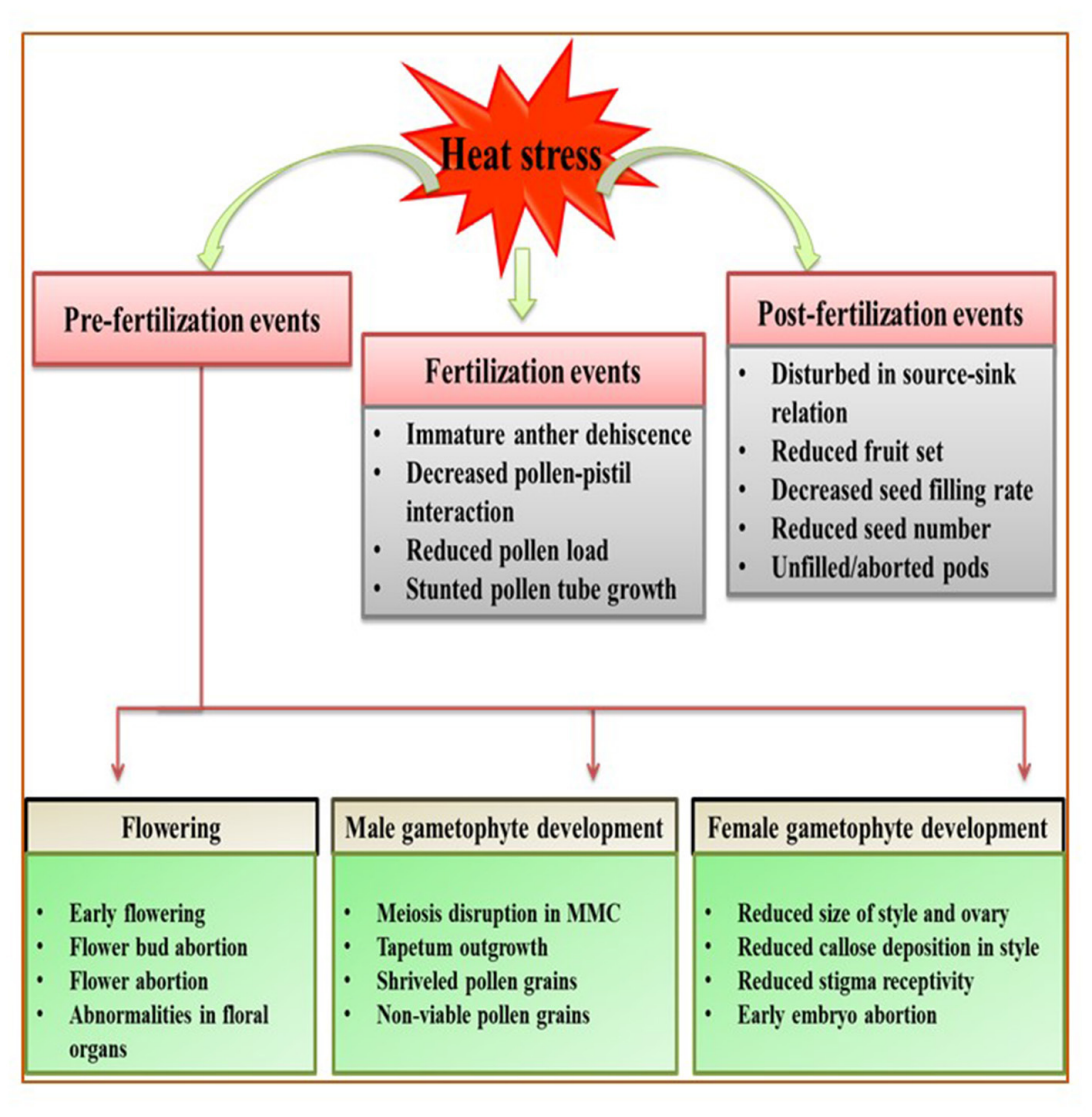
Generalized overview of the effects of heat stress (HS) on the reproductive stage of plants, broadly categorized into three events: pre-fertilization, fertilization, and post-fertilization. Heat stress affects the flowering stage by promoting early flowering and flower bud/flower abortion. During male gametophyte development, heat stress disrupts meiosis and decreases tapetum growth, resulting in shriveled and non-viable pollen grains. During female gametophyte development, heat stress reduces style and ovary size and callose deposition, reduces stigma receptivity, and causes early embryo abortion. Moreover, immature dehiscence and malformed pollen grains result in poor pollination and fertilization. Heat stress during post-fertilization decreases the seed filling rate and disturb source–sink relations, potentially reducing yield manifold.

Reproductive stage is highly sensitive to HS; even a single degree increase for a few hours can be fatal for proper reproductive growth, contributing to poor yields (Prasad et al., 2017). However, studies on reproductive tissues are difficult to assess because gamete development and fertilization are major events that occur over short periods. Here, we categorize the effects of HS in vegetables during three stages of reproduction: pre-fertilization (flower bud initiation, flowering, male and female gametophyte development), fertilization (pollen dehiscence, pollination, pollen reception by stigma, pollen tube growth and fertilization), and post-fertilization events (fruit/pod set, seed development, seed filling) (Figure 2; Table 3).

**Table 3:** Effect of heat stress on reproductive tissues of some vegetable crops.

**Crop**	**Heat stress**	**Effect**	**References**
**Brassica** (*Brassica napus*)	35/23°C	Reduced *in-vitro* pollen germinability, pollen viability, and thinner pollen tubes with stunted & convoluted morphology.	Young et al., 2004
		Microspore and pollen development are sensitive to heat stress.	Sato et al., 2002
**Bell pepper** (*Capsicum annuum*)	33°C	Pollen development (during megaspore mother cell (MMC) meiosis) is greatly reduced. Reduced pollen viability, reduced anther dehiscence, reduced mature pollen grains, slightly swollen and deformed (affect pollen morphology) and without exine layer.	Erickson and Markhart, 2002
**Broad bean** (*Vicia faba*)	34/26°C	Pollen germination	Bishop et al., 2016
**Broccoli** (*Brassica oleracea* var. *italica*)	35°C	Arrested the development of flower buds	Björkman and Pearson, 1998
**Chickpea** (*Cicer arietinum* L.)	40/25°C	Pollen germination, pollen tube growth Pod set	Devasirvatham et al., 2013
**Common bean** (*Phaseolus vulgaris*)	33/27°C 33/29°C	Anther indehiscence and pollen sterility Degeneration of tapetal cells.	Gross and Kigel, 1994
**Cowpea** (*Vigna unguiculata*)	33/30°C	Another development	Ahmed et al., 1992
**Mungbean** (*Vigna radiata* L.)	>40/28°C	Reduced pollen viability, pollen germination, pollen load, stigma receptivity and ovule viability	Sharma et al., 2016
**Okra** (*Abelmoschus esculentus*)	45°C	Incomplete dehiscence, shrunken pollen, smaller anther sacs, reduced pollen number, pollen viability, and pollen germination.	Hayamanesh, 2018
**Pea** (*Pisum sativum*)	36/24°C	Decreased pollen germination, pollen tube growth, pod length, and seed number per pod.	Jiang et al., 2015
**Soybean** (*Glycine max*)	38/28°C	Decreased *in-vitro* pollen germination.	Djanaguiraman et al., 2013b
**Tomato** (*Lycopersicon esculentum*)	32/26°C	Reduced number of pollen grains, pollen viability, and pollen germination.	Sato et al., 2002
	31/25°C	Reduced number of pollen grains, pollen viability, and pollen germination.	Firon et al., 2006
	29°C	Decreased fruit number, fruit weight/plant and seed number/fruit	Peet et al., 1998

### Pre-fertilization Events

#### Flower Bud Initiation

High-temperature stress causes flower bud abortion and abscission of reproductive organs in many crop species, including tomato (Levy et al., 1978; Pressman et al., 2002; Sato et al., 2002), common bean (Konsens et al., 1991), pea (Guilioni et al., 1997), brassica (Angadi et al., 2000), capsicum (Aloni et al., 2001; Erickson and Markhart, 2002), resulting in severe yield losses. Common bean grown at 32/27°C (from flowering to pod maturity) experienced greater abscission and drop of flower primordia (2–5 mm) and flower buds (>5 mm) than at 27/17°C (Konsens et al., 1991). In capsicum, high-temperature stress (33°C for 120 h) affected flower buds (<2.5 mm) and early pistil development less than stamen development, whereas buds (3–4 mm) during tetrad formation and dissolution were highly sensitive to elevated temperature, leading to pollen sterility (Erickson and Markhart, 2002). Flower and flower bud abscission also occurred in heat-stressed (35/15°C for 7 days at early stage) brassica species (Angadi et al., 2000). HS (32/28°C) severely affected flower initiation and development in tomato (Levy et al., 1978; Sato et al., 2002). HS (32/26°C for 8 days before anthesis) in capsicum reduced and altered sucrose mobilization and utilization by flower buds and flowers, resulting in fruit drop and abscission and thus reducing yield by 17% compared to normal sown (28/22°C) (Aloni et al., 2001).

#### Flowering

HS during flowering reduces flower numbers by damaging flower organs, reducing yield (Morrison and Stewart, 2002). HS also decreases the number of flowering branches and thus flower numbers per plant (Harsant et al., 2013). Damage to flower organs has been reported in many crops, including chickpea (Tickoo et al., 1996), common bean (Suzuki et al., 2001; Omae et al., 2012), and mungbean (Kaur et al., 2015). Early flowering and flower abortion are other impacts of HS, as reported in pea (Guilioni et al., 1997), tomato (Sato et al., 2004), common bean (Omae et al., 2012), and mungbean (Sharma et al., 2016).

#### Male Gametophyte Development and Function

Threshold temperatures needed to impose damages in reproductive tissues are less than the one needed to cause injury to vegetative tissues. Male gametophytes are more sensitive to HS than female gametophytes, with lower threshold temperatures than vegetative tissues. HS damage can occur pre-pollination or post-pollination, impairing fertilization and ultimately reducing seed set (Sage et al., 2015). Pre-pollination events that are highly susceptible to high temperature are (1) meiosis I and meiosis II of the microspore mother cell (Young et al., 2004), (2) development and subsequent dissolution of the tapetum layer (Farooq et al., 2017), and (3) exine and intine formation (Nahar et al., 2016). Post-pollination events affected by HS are (1) pollen load, (2) pollen germination, (3) pollen tube growth, and (4) fertilization (Hedhly et al., 2009; Sita et al., 2017). The sensitivity of male gametophytes to HS varies according to plant species (Li et al., 2018).

HS reduced fertility of microgametophytes in brassica (Rao et al., 1992) and impaired meiosis in tomato, damaging pollen germination and pollen tube growth (Foolad, 2005). In soybean, HS reduced pollen production, germination, tube elongation, and impaired pollen development (no apertures and disturbed exile ornamentation) (Salem et al., 2007; Nahar et al., 2016; Djanaguiraman et al., 2019). In capsicum, HS produced shrunken and empty microspores without an exine layer (Erickson and Markhart, 2002). Shriveled pollen grains under HS may be due to decreased starch accumulation in anther walls and pollen grains reducing soluble sugars for their development (Pressman et al., 2002).

#### Female Gametophyte Development and Function

Female gametophytes are relatively more tolerant to HS than male gametophytes (Hedhly, 2011). HS impairs megaspore mother cell development by impeding meiosis, reducing pistil size, reducing stigma receptivity due to poor pollen adhesion, reducing stigmatic papillae for holding pollen grains, interrupting nutrient transport from style to pollen impeding pollen tube germination and growth, as noticed in chickpea (Kaushal et al., 2016), bean (Porch and Jahn, 2001) and cowpea (Ahmed et al., 1992). HS, reduced callose deposition in lentil styles (Bhandari et al., 2017), reduced the amount of attractants from ovule synergids cells that misguide the pollen tube (Saini et al., 1983) to severely affect the fertilization. Furthermore, HS damages the embryo sac and causes early embryo abortion, likely arresting fertilization; for instance, in tomato, HS exposure (40°C for 3 h) for 4 days before anthesis resulted in aborted embryos with degenerated eggs and synergids (Iwahori, 1965). Abnormalities in embryo sac development have also been observed in brassica, reducing seed set and yield (Polowick and Sawhney, 1988). HS also reduced ovule viability in common beans (Ormrod et al., 1967; Suzuki et al., 2001). Unlike, male gametophyte, detailed impacts of HS on female gametophyte organs are, however, barely known. This may be because of the reason that female gametophyte is protected inside the ovary and sheltered and difficult to reach and dissect.

### Fertilization

High-temperature stress (>30°C) negatively impacts male and female gametophyte development, leading to poor development and deformities of reproductive tissues, limiting the fertilization process in many plant species (Saini and Aspinall, 1982; Prasad et al., 2017). HS also reported to affect the flower pollination rate in tomato resulting in low fruit set with reduced lycopene content and fruit quality (Alsamir et al., 2021) Indehiscent anthers, non-viable pollen, and poor stigma receptivity are possible causes for fertilization failure and sterility imposition in many crops, including chickpea (Kumar et al., 2013), soybean (Board and Kahlon, 2011), mung bean (Kaur et al., 2015), tomato (Pressman et al., 2002), common bean (Porch and Jahn, 2001), and capsicum (Erickson and Markhart, 2002).

### Post-fertilization Events

#### Fruit/Pod Set

High-temperature stress affects the proportion of flowers forming fruits (fruit set) (Prasad et al., 2000). HS (38/30°C) markedly decreased fruit weight (51.6%), fruit diameter (25%), fruit length (30%), and seed number per fruit (57%) in sweet pepper compared with normal temperature (33/21°C) (Thuy and Kenji, 2015). Peet et al. (1998) reported that high temperature (29°C) decreased fruit number (10%), total fruit weight/plant (6.4%) and seed number/fruit (16.4%) in male fertile tomatoes compared to optimum temperature (25°C). The high temperature impaired pollen development and release, leading to reduced fruit set in male-fertile tomatoes compared with male-sterile lines. Similarly, fruit set and fruit size in tomato plants declined at 29/23°C compared to 24/18°C (Saha et al., 2010). HS seriously damaged fruit set in tomatoes exposed to 40°C for 4 h before anthesis and reduced the pollen germination from 79.5% (at 30/17°C) to 30% and pod set from 63% (at 30/17°C) to 14.9% (Rudich et al., 1977). In Common bean, high temperature (32/27°C) reduced the pod set from 17 to 97%, seed set by 39–98%, and seeds/pod by 42 to 73% compared to control temperature (22/17°C) (Gross and Kigel, 1994). Similar finding on bean plants exposed to even higher temperatures (40/30°C) had fewer filled pods, parthenocarpic pod development, sickle-shaped pods, reduced seed size, and fewer seeds/pod and total seeds than control condition (Prasad et al., 2002; Soltani et al., 2019). In peas, high temperature (32°C for 6 h) at the reproductive stage increased the abortion rate of reproductive organs (flower buds and young pods) from 20 to 50% which reduce seed yield (Bueckert et al., 2015).

#### Seed Development and Seed Filling

Seed formation and seed filling are the last phases of the life cycle of seed plants; and; HS drastically affects seed development and the seed-filling phase, increasing the fraction of abnormal and shriveled seeds (Sehgal et al., 2018). In common bean, a linear relationship between temperature and grain weight was recorded resulting in a significant decrease in seed weight, i.e., 0.07 g per °C when temperature was raised beyond 31/21°C (Prasad et al., 2002). Seed development starts from cell division and, when seed cells are fully formed, storage reserves start to accumulate (Egli, 1998). Direct effects of HS on division and size of endosperm cells are well-documented (Commuri and Jones, 2001). Reduced division and size of endosperm cells results in accumulation of fewer carbohydrates, proteins, lipids, and starch accumulate in developing seeds. HS also accelerates the rate and duration of seed filling, resulting in abnormal seeds and significant yield losses (Farooq et al., 2017). Not only yields, HS affects seed quality characteristics, reducing seed number and size, degrading nutrient composition, and decreasing seed viability, through impaired nutrient uptake, assimilate partitioning, and translocation (Prasad et al., 2008). Starch, proteins, and lipids are the principal reserves transferred from the main plant to developing seeds (Alencar et al., 2012), but HS limits their synthesis and translocation during seed filling, affecting grain quality (Farooq et al., 2017), and could be due to decreased enzyme activity. The activity of starch synthesizing enzymes, such as starch synthase, sucrose synthase, and invertase, decrease under HS, as reported in pea (Smith and Denyer, 1992) and chickpea (Kaushal et al., 2013). Similarly, HS disrupts seed storage proteins, such as β-glycocynin and globulin 11S in soybean (Hashizume and Watanabe, 1979; Iwabuchi and Yamauchi, 1984), and sucrose-synthesizing enzymes and proteins that aid in sucrose translocation. Reduced sucrose synthase activity affects the sucrose and starch ratio, decreasing the transfer of soluble carbohydrates to developing ovules, as reported in pea (Jeuffroy et al., 1990) and cowpea (Ismail and Hall, 1999). Reduced crop duration and seed filling has been correlated with an inefficient light capture ability (canopy growth rate) in small plants, decreasing the photosynthetic rate and thus seed size, as reported in soybean (Board and Kahlon, 2011). Prasad et al. (2002) reported a linear relationship between temperature and grain weight in common bean, with seed weight decreasing by 0.07 g per °C at temperatures above 31/2.

## Physiological Aspects and Cellular Functions Under Heat Stress

### Membranes

**Figure 3:**
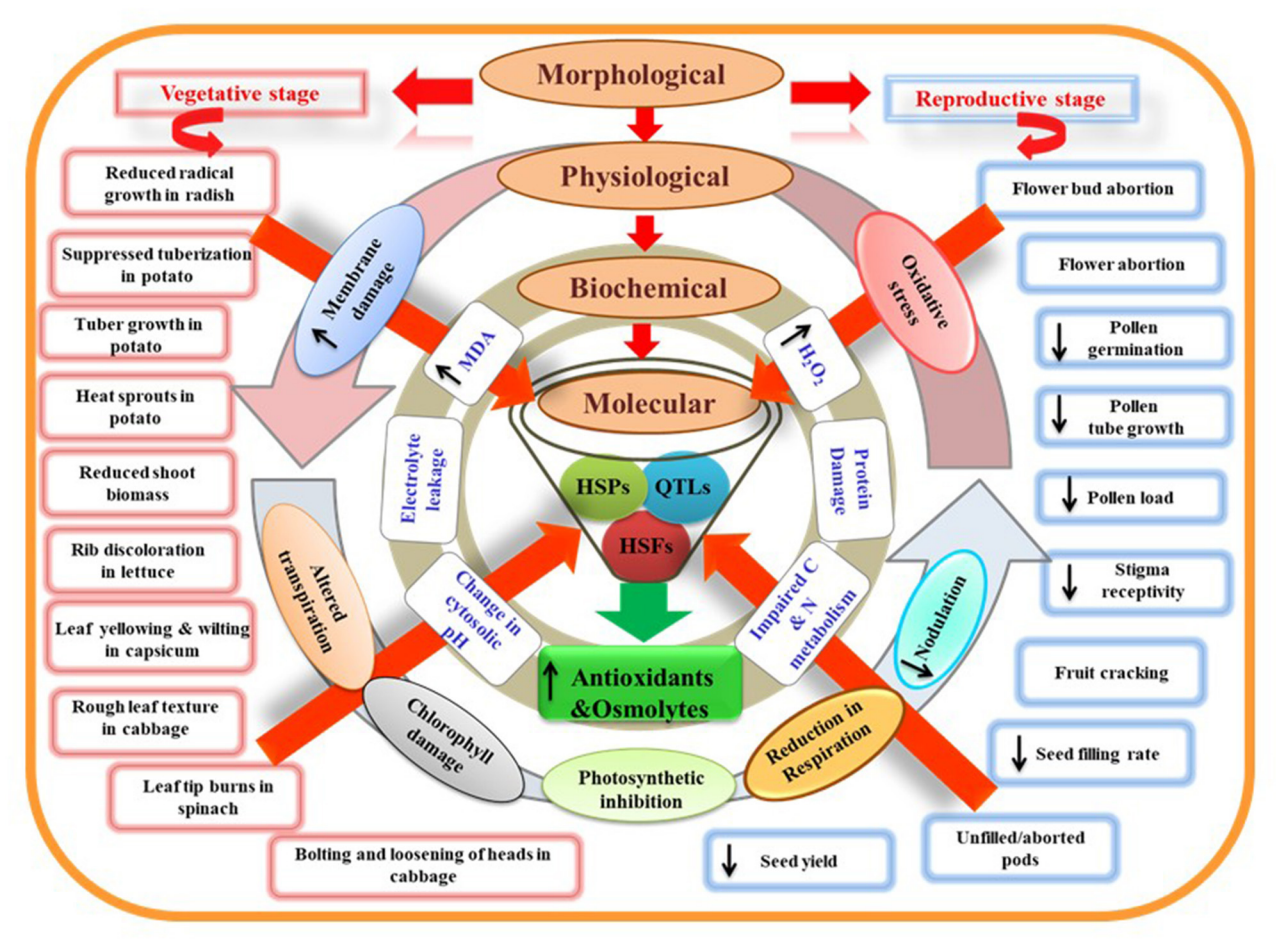
Model representing morphological, physiological, biochemical, and molecular characteristics of plants under heat stress. Morphological damages at vegetative and reproductive stages can be visualized as direct measures of plant stress. At the physiological level, these damages are associated with leaky plasma membrane, altered transpiration, chlorophyll damage, reduced photosynthesis, respiration, and nodulation rate. Disturbed physiological processes can promote oxidative stress damage observed through stress indicators like increased *malondialdehyde* (*MDA*) and hydrogen peroxide (H_2_O_2_) content. Protein damage and impaired carbon and nitrogen metabolism due to impaired enzymatic activities further exaggerate stress levels at the biochemical level. Heat shock proteins (HSPs), heat shock factors (HSFs), and quantitative trait loci (QTLs) related to heat stress responses of plants may play a key role in the plant adaptation. HSPs and HSFs have a central role in regulating the activity of various genes that amplify the production of antioxidants and osmolytes and are helpful governing thermotolerance.

HS disrupts the organization of the plasma membrane by increasing unsaturated fatty acids, thus making the membrane more fluid (Hofmann, 2009), and influencing the cellular functions by initiating a signal cascade (Firmansyah and Argosubekti, 2020; Hassan et al., 2021). HS also accelerates the kinetic energy and movement of various molecules through the membrane. Further, protein denaturation and altered tertiary and quaternary structure of membrane proteins increase membrane fluidity (Savchenko et al., 2002). Thus, HS disturbs primary processes of plant-like photosynthesis and respiration due to increased permeability or solute leakage from cells (Figure 3). Therefore, cell membrane thermostability trait used to evaluate HS on plants and identify heat-tolerant and heat-sensitive genotypes; for example, in soybean (Martineau et al., 1979), potato (Chen et al., 1982), and cowpea (Ismail and Hall, 1999). The effectiveness of cell membrane thermostability assays depends on the tissue type and stress type used for plant adaptation. It is also unknown whether membrane thermostability is linked to other plant characteristics that confer heat tolerance, such as growth and yield.

### Photosynthesis

Photosynthesis is highly sensitive to HS and photosynthetic activity reduces drastically under HS. Studies have detailed the affected photosynthetic mechanisms that ultimately reduce the photosynthetic capacity of plants (Berry and Bjorkman, 1980; Sharkey, 2005). Thylakoid reactions, Rubisco activity, and photosynthetic pigments are generally disturbed by HS. HS primarily affects the physical state and structure of the thylakoid membrane by triggering thylakoid leakiness and unstacking thylakoids, damaging the D1 protein of PSII (Sharkey, 2005). To counterbalance these reactions, zeaxanthin synthesis increases, affecting the normal state of thylakoids (Havaux, 1996). HS disturbs the electron flow between the two photosystems (PSI and PSII) and reduces the photosynthetic efficiency of plants. HS also accelerates the phosphorylation of light-harvesting complex (LHCII) and disconnects it from PSII core complex, thus decreasing its turnover rate, but increasing the turnover rate of PSI (Wise et al., 2004). HS dephosphorylates core proteins (D1, D2, and CP43), deactivating PSII (Yamamoto et al., 2016). HS alters the fluorescence induction parameters, measured as the Fv/Fm ratio; this ratio helps to determine the quantum efficiency of PSII and indicates the rate of linear electron flow and overall photosynthetic performance of plants (Jamil et al., 2007). HS decreased chlorophyll a fluorescence, PII quantum yield, photochemical quenching, and increased respiration rate in soybean (Djanaguiraman et al., 2013a).

Along with thylakoid reactions, HS triggers the deactivation of Rubisco (Crafts-Brandner and Salvucci, 2000). Rubisco being dual enzyme catalyses the carboxylation of ribulose−1-5-bisphosphate in the photosynthetic Calvin cycle and oxygenation in the photorespiratory pathway; the ratio between two reactions governs the photosynthetic efficiency of plant. But the elevated temperature inhibits the CO_2_ fixation and increases the oxygenase activity and reduces photosynthetic rate (Crafts-Brandner and Salvucci, 2000). Rubisco activation is not only associated with pH and Mg^2+^ concentration of stroma but also with Rubisco activase (RA); an ATPase. RA induces the activation of the Rubisco by increasing the proportion of its active sites and brings conformational changes that allow CO_2_ and Mg^2+^ for activation and carbamylation. High temperature can disturb the pH and Mg^2+^ concentration of stroma, interfering with the carbamylation step of Rubisco activation (Weis, 1981a,b) and also caused RA dissociation because of its poor structural stability and heat labile nature (Demirevska-Kepova and Feller, 2004). Few reports have noticed that heat stress affects the photosynthesis through heat sensitivity of Rubisco and RA activity, for instance in tomato, heat stress (40°C for 8 h for 6 days to 3 weeks old plant) decreased the accumulation of Rubisco enzyme's isoforms (Parrotta et al., 2020), as in pea (Haldimann and Feller, 2005), potato (Cen and Sage, 2005) and spinach (Zhao Q. et al., 2018).

Pea plants exposed to HS reduced chlorophyll biosynthesis due to the destruction of various enzymes involved in biosynthetic pathways (Dutta et al., 2009; Aleem et al., 2021). HS decreased the activity of first enzyme of the biosynthetic pathway, 5-aminolevulinate dehydratase, in cucumber (Tewari and Tripathy, 1998). Decreased chlorophyll content, Chl a/b ratio, and chlorophyll/carotenoid ratio have been reported in many crops under HS (Aien et al., 2011) (Table 4). Similarly, HS stress causes pre-mature leaf senescence in soybean leaves which results in decreased photosynthesis primarily due to decreased chlorophyll content, higher reactive oxygen species, lower antioxidants, and increased thylakoid membrane damage (Djanaguiraman and Prasad, 2010). HS increased ethylene production in leaves which was one of the reasons of premature leaf senescence in soybean (Djanaguiraman and Prasad, 2010). Detailed anatomical studies showed that HT stress significantly increased the thicknesses of the palisade and spongy layers and the lower epidermis (Djanaguiraman et al., 2013a). In addition, HT stress damaged the plasma membrane, chloroplast membrane, thylakoid membranes; mitochondrial membranes, cristae, and matrix were distorted which led to decreased photosynthesis (Djanaguiraman et al., 2013a) (Figure 3).

**Table 4:** Effect of heat stress on photosynthesis in some vegetable crops.

**Crop species**	**Temperature**	**Effect**	**References**
**Broad bean** (*Vicia faba*)	42°C	Decreased content of Chl a, Chl b, and carotenoids	Hamada, 2001
**Cabbage** (*Brassica oleracea* var. *capitata*)	40°C	Decrease in F_v_/F_m_ values and photosynthetic efficiency	Chang et al., 2016
**Cauliflower** (*Brassica oleracea* var. *botrytis*)	40°C	Significant reduction in chlorophyll fluorescence F_v_/F_m_ Inhibition of CO_2_ fixation and damage to photosynthetic electron transport at site of PS II	Lin et al., 2015
**Chickpea** (*Cicer arietinum* L.)	40/30°C	Reduced chlorophyll content	Kaloki et al., 2019
**Common bean** (*Phaseolus vulgaris*)	45°C	Partially-reversible inactivation of PS-II and dissociation of light harvesting complex from reaction center of PS-II Destruction of PS-II reaction center and formation of quenching species	Costa et al., 2003
**Cowpea** (*Vigna unguiculata*)	30/25°C	Reduced rate of photosynthesis	McDonald and Paulsen, 1997
**Cucumber** (*Cucumis sativus* L.)	33–48°C	Decline in PS II activity and photochemical quenching Decreased net photosynthetic rate	Ding et al., 2016
	42°C	Chlorophyll biosynthesis	Tewari and Tripathy, 1998
**Mungbean** (*Vigna radiata*)	>40/28°C	Decline in PS II activity	Sharma et al., 2016
**Okra** (*Abelmoschus esculentus*)	>39°C	Adverse effects on the photosynthetic apparatus	Hayamanesh, 2018
**Pea** (*Pisum sativum*)	>40°C	Decreased photosynthetic electron transport Complete suppression of photosynthetic electron transfer	Haldimann and Feller, 2005
	45°C	Decreased CO_2_ assimilation and O_2_ evolution	Georgieva et al., 2000
**Potato** (*Solanum* spp.)	25°C	Decreased photosynthetic rate Decreased Chl a+b and carotenoid content	Aien et al., 2011
	38°C	Rapid and irreversible loss of PS II	Aien et al., 2011
**Soybean** (*Glycine max*)	38/28°C 38/30°C	Decrease in leaf photosynthetic rate by 20.2% Significantly affects net photosynthesis and total chlorophyll content Decreased chlorophyll content, photosynthetic rate,	Nahar et al., 2016
	39/20°C	Severely damaged PSII site	Li et al., 2009
**Spinach** (*Spinacia oleracea*)	40°C	Inhibition of oxygen evolution Cleavage of D1 protein of PSII	Yoshioka et al., 2006
**Tomato** (*Solanum lycopersicum*)	36/38°C	Decreased F_v_/F_m_ values and PS II damage Decreased net photosynthetic rate Decreased chlorophyll content	Zhou et al., 2017

### Nitrogen Content, Fixation and Nodulation

Nitrogen is one of the main nutrients required by the plant for proper growth, development and productivity. It is the constituent of various important organic compounds like amino acids, proteins, nucleic acids, enzymes, and the chlorophyll molecule (Christophe et al., 2011). Nitrogen content in the plant measured as nitrate, ammonium ions, and proteins. Besides performing basic roles in plants, its metabolism is also very crucial for heat tolerance because it increases the osmolyte content and antioxidant enzyme activity (Ru et al., 2022). Studies have also shown their role in promoting the HSP production (Heckathorn et al., 1996). Osmolytes like proline and quaternary ammonium compounds, being nitrogen rich and accumulate in plants under heat stress conditions (Rivero et al., 2004). Ammonium ion and proline accumulation confer heat tolerance to tomato and promoting higher biomass production (Rivero et al., 2004). During the reproductive period, nitrogen concentration successively increases when temperatures rise for example in pea, when high temperature occurs during or after flowering seed N concentration is increased (Larmure et al., 2005). Similarly, in soybean, seed N concentration increases during the reproductive period at temperature 40/30°C (Thomas et al., 2003). Increases in the accumulation of proteins; level of globulin protein storage causing a reduction of the albumin/globulin content in mature seeds (Hurkman et al., 2009). In pea, the final level of vicilin storage proteins was higher under heat stress (Bourgeois et al., 2009). However, in tomato roots, it has been reported that HS disturbs enzymes involve in nitrogen metabolism (nitrate and ammonium assimilation) thereby decreasing total protein content and level of nutrient uptake and assimilation (Giri et al., 2017). Further, studies on the contrasting genotypes of brassica revealed that HS (40/30°C for 7 days) negatively affected the activities of nitrogen assimilation enzyme including Glutamate synthase (GOGAT), glutamine synthetase (GS), glutamate dehydrogenase (GDH), more in heat sensitive genotype (WS-6) as compared to heat tolerant genotype (WS-1). These enzymes help in possessing better photosynthetic nitrogen use efficiency (Yuan et al., 2017).

Symbiotic nitrogen fixation in leguminous crops depends on the presence of appropriate *Rhizobium* species in the vicinity of root zone, however, almost all processes starting from rhizobial survival to host infection and nitrogen fixation depend mainly on the environmental factors, such as soil temperature (Bordeleau and Prévost, 1994). High temperature interferes with almost all processes of symbiotic nitrogen fixation, directly as well as indirectly, soil temperature affects not only the rhizobial survival in the root zone but also the exchange of molecular signals between two symbiotic partners (Alexandre and Oliveira, 2013). Rhizobial strains have an optimum soil temperature (25–30°C) for their growth and nitrogen fixing ability and Rhizobia are greatly affected by high soil temperature. However, optimum temperature varies with the crop species, for instance, in soybean, weak rhizobia were formed at 40°C and no rhizobia were isolated at 45°C (Chen et al., 2002). HT interferes directly with nodule development as it hampers nodule development and increases nodule senescence (Aranjuelo et al., 2007). HS affects indirectly the nitrogen fixation by inhibiting the formation of root hairs, infection thread formation, reducing the nodulation sites, adherence between bacteria and root hair (bacterial infection), and bacteroid formation (Zahran, 1999; Hungria and Vargas, 2000; Alexandre and Oliveira, 2013).

Elevated temperature also affects nodule growth rate, nodule size, and nodule fixation ability, as reported for common bean exposed to HS (35 and 38°C/8 h/day) at the flowering stage (Hungria and Franco, 1993). Another study showed that at 47°C temperature no nodules were formed in common bean (Karanja and Wood, 1988). Studies have shown that nodulation ability varies inversely with temperature, and legume species differ in their temperature endurance; for instance, common bean is more sensitive to temperature stress than cowpea and soybean for nitrogen fixation (Piha and Munns, 1987). In cowpea, the optimum temperature for nodule growth and development is 30–36°C; temperatures above 40°C lead to fewer or no nodules (Day et al., 1978). In common bean, nodules that formed at high temperature (≥35°C) were inefficient and unable to fix nitrogen (Hungria and Franco, 1993). Piha and Munns (1987) noted that nodules formed at 35°C were small and had low nitrogenase activity. The optimum temperature for nodule growth is 20°C for pea and 25–30°C for soybean (Michiels et al., 1994). HS decreased nodulation ability in mungbean (Sharma et al., 2016). In common bean, HS affected nitrogen fixation due to decreased activity of enzymes involved in nitrogen metabolism, such as dinitrogenase complex, glutamine synthetase (GS), and glutamine synthase (GOGAT), decreasing the concentration of ureids-N in nodules and xylem sap (Hungria and Kaschuk, 2014). Prasad et al. (2000) observed that high soil temperatures (35°C) significantly decreased number of nodules and nodule dry weight per plant compared to optimum soil temperature (25°C) in peanut.

**C.N ratio:** Plant growth and defense are both fuelled by compounds synthesized from a common pool of carbon and nitrogen, implying the existence of a competition for carbon and nitrogen allocation to both metabolisms. The ratio of carbon to nitrogen (C: N) of an organ is often regarded as a convenient indicator of growth and quality. Almost a century ago, plant nutrition was considered a crucial factor in controlling flowering time. According to Klebs (1913), a high endogenous carbon: nitrogen ratio promotes flowering, while a low carbon: nitrogen ratio promotes vegetative growth. Inferred from the fact that (a) conditions favoring photosynthetic CO_2_ fixation generally accelerate flowering and (b) high nitrogen intake (fertilizers) might delay or reduce reproductive development in some plants (Bernier et al., 1981). The flowering percentage increased when NH_4_NO_3_ concentration decreased from 16.5 to 8 g l^−1^, in tomato plant (Dielen et al., 2001). Royer et al. (2013) revealed that C:N ratio in the pool of resources in the total plant, were correlated with the concentrations of diverse compounds of the primary and secondary metabolisms in young tomatoes. Under HS, Peet et al. (1997) found that in tomato plants, the carbon and nitrogen metabolism get imbalanced, and stem and petiole elongation consume too much nutrients, which in turn reduces the dry matter storage of the plant, affecting tomato quality and yield. Soil mixed with dry powder of *Sesbania* plant (leaves + tender stems; C: N ratio 15.4) plays effective role in enhancing resistance and resilience (stability) of soil microbial activity against heat stress (Kumar et al., 2014). Heat stress may accelerate leaf senescence and increase respiration rate which consequently decreases plant N and C availability for seeds and shorten the duration of seed filling period in soyabean (Egli and Wardlaw, 1980). Thus, balanced C:N ratio plays an important role in plant physiological process. Similarly, Larmure et al., 2005 demonstrated that the lower seed N concentration in pea plant at the average temperature range (13–23°C) can be explained by prolonged duration of the seed-filling associated with the lower seed N concentration, higher C availability for the seeds. Because the rate of seed N accumulation per degree-day mainly depends on N availability to seed filling, the rate of N accumulation was higher at 25/20°C than at lower temperature. HS reduces seed size and modifies the C:N ratio in the period of seed formation in pea (Guilioni et al., 2003).

### Antioxidants and Oxidative Stress

Severe HS generates ROS, such as hydrogen peroxide (H_2_O_2_) and superoxide radical (${\text{O}}_{2}^{-}$), as byproducts of the aerobic metabolism, which adversely affect cellular metabolism, such as lipid membrane peroxidation, and damage nucleic acids and proteins (Bita and Gerats, 2013). Plants respond to ROS production by activating enzymatic and non-enzymatic ROS scavenging systems (Bita and Gerats, 2013). The main ROS scavenging enzymes are superoxide dismutase (SOD), catalase (CAT), peroxidase (POX), ascorbate peroxidase (APX) glutathione reductase (GR), whereas non-enzymatic chemical are ascorbic acid (ASC) and glutathione (GSH) (Suzuki et al., 2012). SOD helps scavenge ${\text{O}}_{2}^{-}$ whereas CAT and POX degrade H_2_O_2_. Elevated levels of these antioxidants are crucial in imparting thermotolerance in plants (Awasthi et al., 2014). In soybean, ROS accumulation (mainly H_2_O_2_ and ${\text{O}}_{2}^{-}$) due to HS is associated with decreased enzyme activities of various antioxidants (Djanaguiraman et al., 2005, 2013a). Similarly, GR and CAT activities decreased in common bean under oxidative stress (Babu and Devaraj, 2008). Likewise, decreased APX and GR expression occurred in mungbean exposed to HS (Sharma et al., 2016). However, relationship between antioxidant enzymes and HS is far more complex in tomato where activity of SOD, APX increased and CAT activity decreased (Zhou et al., 2014). This complexity was also evident in capsicum where, NADPH oxidase and CAT activity increased at high temperature (Gulen et al., 2012). In chickpea, tolerant genotypes had higher SOD, CAT, APX, and GR activity than sensitive genotypes under HS (40/30°C and 45/35°C) (Kumar et al., 2013). Moderate HS increases the expression of various enzymatic antioxidants, while severe HS suppresses it (Wilson et al., 2014).

## Defense Responses

In addition to antioxidants, plants endure HS by activating major defense mechanisms which are mainly comprised of increased production of heat shock proteins (HSPs) and compatible solutes (Sakamoto and Murata, 2002; Wahid et al., 2007; Mittler et al., 2012; Khan and Shahwar, 2020). HSPs are the molecular chaperones that protect the misfolded proteins from irreversible aggregation, sorting, translocation, and degradation, important for establishing cellular homeostasis in normal and stressed conditions (Vierling, 1991). There are five classes of HSPs categorized according to their molecular weight: HSP100, HSP90, HSP70, HSP60, and Small HSP (sHSP), and located in the cytoplasm as well as cellular orgenelles, nucleus, chloroplast, mitochondria, and endoplasmic reticulum (Wang et al., 2004). Different chaperone families though have a peculiar role but coordinate cellular homeostasis. Chaperones also maintain crosstalk with signaling molecules, antioxidants (acerbate peroxidase), and osmolytes (trehalose, proline, glycine betaine) (Wang et al., 2004; Kang et al., 2022). Various reports have confirmed accumulation of all HSP families in different vegetables and food legumes under HS, with greater accumulation of sHSPs than other HSPs, as reported for spinach (Guy and Li, 1998), tomato (Preczewski et al., 2000), soybean (Ortiz and Cardemil, 2001), common bean and cowpea (Simões-Araújo et al., 2003), potato (Ahn et al., 2004), cabbage (Park et al., 2013), pea (Talalaiev and Korduym, 2014), faba bean (Kumar et al., 2015), capsicum (Li et al., 2015), chickpea (Meena et al., 2017), and broccoli (Lin et al., 2019). Accumulation of these proteins helps plants to re-establish homeostasis under HS conditions. Hence, the expression level of HSPs and HSFs could be manipulated genetically to improve heat tolerance ability. Overexpression of HSPs facilitates transformed cells to endure HS better than non-transformed cells (Grover et al., 2013); for instance, overexpression of sHSP (HSP21) in transgenic tomato imparts stable PSII, shielding photosynthesis from temperature-dependent oxidative stress and accumulating more carotenoids under HS (Neta-Sharir et al., 2005). Furthermore, overexpression of HSFs facilitates the expression of HSPs; for example, overexpression of HSFA1 in transgenic soybean enhanced the expression of GmHSP70 leading to thermotolerance (45°C) (Zhu et al., 2006). Similarly, overexpression of transcription factor (CaWRKY40) enhanced thermotolerance in capsicum (Dang et al., 2013).

The role of various osmolytes, including proline and glycine betaine, in imparting heat tolerance is well-documented (Sakamoto and Murata, 2002). Osmolytes are low molecular weight compounds that can buffer cellular redox potential under HS. Proline is a well-studied osmolyte, concentration of which increases by several-fold under stress conditions. A heat-tolerant cabbage genotype accumulated more proline (and soluble sugars and antioxidants) than a sensitive genotype (Song et al., 2019). Similarly, Paul et al. (2014) even suggested using increased proline and soluble sugars in potato under HS can used as markers for selecting heat-tolerant genotypes. Increasing HS gradually increased proline and soluble sugar contents in lettuce seedlings, indicating heat tolerance (Han et al., 2013). The role of proline in thermotolerance was also confirmed using exogenous proline applications. Kaushal et al. (2011) noted that exogenous treatment of proline induced thermotolerance in chickpea by protecting the enzymes involved in carbon and antioxidant metabolism. Glycine betaine is another compound that confers heat tolerance; Aien et al. (2011) suggested that glycine betaine imparts heat tolerance in potato genotypes under HS conditions.

### Heat Avoidance

Heat avoidance through transpiration cooling is the best strategy adopted by plants to minimize the losses (Julia and Dingkuhn, 2013) Under moderately HS conditions, plants can accelerate growth to promote plant thermonastic responses and architectural changes to move susceptible parts away from soil heat flow or to improve evaporative cooling (Havko et al., 2020). In soybean, tomato, or cabbage, moderately high ambient temperature induces hypocotyl elongation, and tomato displays leaf hyponasty (Quint et al., 2016; Casal and Balasubramanian, 2019; Vu et al., 2019). Pea canopies architecture and leaf type as traits of heat resistance can avoid heat and maintain a lower canopy temperature as leafed cultivars have greater leaf surface area and likely greater transpirational cooling, assuming soil moisture availability and an adequate root system (Tafesse et al., 2019). Another study showed that the leaf movement capacity in beans was shown to function in direct sunlight avoidance and benefited the plant by protecting it against photoinhibition and by maintaining leaf temperatures lower than the air temperature (Pastenes et al., 2004). Thus, as novel donors with higher heat tolerance or escape provides, there is an ample evidence for systematic exploration of wild species and accessions (Prasad et al., 2017) for introducing these traits.

## Identification of Tolerant Genotypes and Improving Adaptation and Mitigation to HS

### Physiological Approaches

Heat tolerance is a polygenic trait greatly influenced by environmental changes (Blum, 2018). HS effects are stage-specific, with the response at one stage differing from the response at another. Breeders employ various techniques to minimize the impact of an unpredictable environment on crops. Conventional breeding is the oldest but most prevalent method, primarily based on selecting phenotypic plant characters (Acquaah, 2015). In recent decades, new techniques have emerged based on morpho-physiological plant characters merged with conventional breeding methods to screen superior varieties. These methods exploit inbuilt plant properties to cope with HS and assist in selecting heat-tolerant genotypes. Screening germplasm of various vegetable crops using various physiological traits linked to heat tolerance would be useful for breeding programs focused on developing HS tolerant genotypes. Although there are several methods or traits used for screening, some of the most common are discussed.

### Stay-Green Assay

The stay-green character is the plant's ability to retain chlorophyll and remain green for longer to sustain photosynthesis, especially during seed filling (Thomas and Howarth, 2000). However, the adverse impacts of HS cause leaves structural changes and chlorophyll degradation and it ultimately induces premature, leaf senescence (Djanaguiraman and Prasad, 2010; Jha et al., 2014). Moreover, the onset of HS during seed filling affects various physiological processes, including increased leaf senescence (chlorophyll loss), altered source–sink relationship, and decreased assimilation of reserve food material in developing seeds, limiting plant yield (Luche et al., 2015). Therefore, delayed leaf senescence may be associated with heat tolerance, enabling plants to maintain their photosynthetic ability (Lim et al., 2007). High chlorophyll and carotenoid contents in leaves improve the photochemical efficiency of plants and reduces ROS concentration in plants such as tomato (Zhou et al., 2015) and pea (Tafesse, 2018).

In addition, the stay-green character positively correlates with canopy temperature depression. Stay-green genotypes have lower canopy temperatures due to transpirational cooling than non-stay-green genotypes (Kumari et al., 2013). In addition to these modifications, HS also causes plant morphological and architectural modifications like leaf hyponasty (measured through leaf angles), leaf petiole elongation, small and thin leaves, that are helpful for the plants to keep their canopies cool. For instance, the cucumber species have hyponastic leaves (Park et al., 2019) and reduced leaf size is found in potato (Tang et al., 2018) and capsicum species (Utami and Aryanti, 2021) under heat stress conditions. These processes involve various signaling cascades that mediate the developmental shaping for environment adaptation in plants (Gil and Park, 2019). This trait is also associated with grain yield and quality and abiotic stress tolerance (Kamal et al., 2019). Hence, the stay-green trait is essential for improving crop yield and useful for imparting heat tolerance (Joshi et al., 2007; Kusaba et al., 2013), and thus may be an important genetic trait for improving crop yield under HS.

### Canopy Temperature Depression

Canopy temperature depression (CTD) is usually measured as the difference between air and canopy temperature, indicating the plant's ability to lower its foliar temperature by transpirational cooling, as measured by an infrared thermometer. CTD also reflects plant water status and is influenced by the plant's ability to extract water and the transpiration difference between air and plant. Accordingly, CTD has been used to select heat-tolerant and drought-tolerant genotypes. Plants that can maintain cooler canopies during seed filling can tolerate high-temperature stress (Munjal and Rana, 2003). Heat-tolerant varieties of capsicum (Gajanayake et al., 2011) have been selected based on the stay-green trait. In soybean, there is a direct relationship between CTD, canopy greenness, photosynthetic rate, and yield (Kumar et al., 2017). Thus, the CTD trait can be used as a critical genetic trait for crop improvement aimed at increased yields at the vegetative stage.

### Cell Membrane Thermostability

HS is amounts of sensed by cell membranes of leaf tissues, weakening cell membrane integrity/rigidity due to an increased degree of unsaturated fatty acids that increase membrane fluidity. This may change membrane permeability and disturb the selective transport of molecules across the membrane, affecting cellular homeostasis (Marcum, 1998). HS can directly affect membrane integrity through photochemical modifications during photosynthesis or ROS (Bita and Gerats, 2013). Cell membrane thermostability (CMT) can be evaluated with an electrolyte leakage test for screening crops for heat tolerance. The method is simple, quick, and inexpensive compared with whole-plant screening and can be used to assess plant tissue responses at the vegetative stage (Yeh and Lin, 2003). Electrolyte leakage is measured using a conductivity meter, with higher conductivity values indicating higher membrane damage (Nyarko et al., 2008). The CMT test has been used to screen heat-tolerant varieties of many crops, including soybean (Martineau et al., 1979), potato (Nagarajan and Bansal, 1986), cowpea (Ismail and Hall, 1999), cabbage (Nyarko et al., 2008), cauliflower (Aleem et al., 2021) chickpea (Kumar et al., 2013), mungbean (Sharma et al., 2016), and cucumber (Ali et al., 2019).

### Chlorophyll Fluorescence

Chlorophyll fluorescence—expressed as the Fv/Fm ratio (Fv: variable fluorescence; Fm: maximum fluorescence)—is used to detect the state of PSII function in terms of the energy absorbed by PSII in chlorophyll and damage to photosynthetic apparatus by excess light *in vivo* (Maxwell and Johnson, 2000). Chlorophyll fluorescence is a rapid, reliable, and inexpensive procedure for predicting photosynthetic performance under HS. Reduced Fv/Fm values indicate damage to the light-harvesting complex (Moradpour et al., 2021). Chlorophyll fluorescence has been used to select heat-tolerant varieties of sweet pepper (Hanying et al., 2001), common bean (Stefanov et al., 2011), chickpea (Kaushal et al., 2013), mungbean (Kaur et al., 2015), tomato (Zhou et al., 2015; Poudyal et al., 2018), and okra (Hayamanesh, 2018). Makonya et al. (2019) showed that tolerant chickpea genotypes maintain higher Fv/Fm during HS than sensitive genotypes, and Fv/Fm positively correlates with grain yield in the field. Killi et al. (2020) reported the retention of PSII function at elevated temperature positively correlated with antioxidant activity, confirming the applicability of this trait for selecting heat-tolerant varieties.

### Relative Water Content

Relative water content indicates the hydration status of plants and reflects the balance between leaf water supply and transpiration rate. Hence, it can measure leaf water deficit and the degree of damage under HS (Mullan and Pietragalla, 2012). High transpiration increases water loss, which can cause tissue dehydration and wilting (Mazorra et al., 2002). Therefore, genotypes that can maintain turgid leaves will minimize HS effects and have numerous physiological advantages. Gowda et al. (2011) suggested using RWC as selection criteria for improving yield under HS. High temperature (40–42°C) at the vegetative and reproductive stage gradually reduced the RWC of capsicum genotypes, more so at the reproductive stage (Puneeth, 2018). RWC has been used to select heat-tolerant genotypes of mungbean (Sharma et al., 2016), capsicum (Puneeth, 2018), common bean (Chavez-Arias et al., 2018), lentil (Sita et al., 2017), tomato (Zhou et al., 2018), cucumber (Ali et al., 2019), and potato (Handayani and Watanabe, 2020) where genotypes with high RWC under HS were rated as heat tolerant.

### Stomatal Conductance

Stomatal conductance measures the rate of carbon dioxide entering or water vapor exiting stomata. This change in transpiration rate facilitates changes in leaf temperature and water potential (Farquhar and Sharkey, 1982). Leaf stomatal conductance is often recognized as an important trait for evaluating differences in response to changing environments. It can be used to determine trait such as photosynthetic CO_2_ uptake, leaf temperature, and water loss (Vialet-Chabrand and Lawson, 2019). Decreased stomatal activity under a changing environment can significantly affect plant growth and biomass (Way and Pearcy, 2012). *In vivo* stomatal conductance can be measured with a steady-state leaf porometer and gas exchange. HS increases *in vivo* adaxial stomatal conductance relative to the control (Sharma et al., 2016). Low stomatal responses under stress can limit photosynthetic rate and cause unnecessary transpiration, decreasing plant water use efficiency and productivity (Matthews et al., 2018). This phenomenon has been used to select heat-tolerant genotypes of sweet pepper (Hanying et al., 2001); tomato (Camejo et al., 2005; Abdelmageed and Gruda, 2009), chickpea (Kaushal et al., 2013), and mungbean (Kaur et al., 2015). While many studies have successfully used one of the traits above to select heat-tolerant genotypes, combining multiple traits would reflect heat tolerance better than relying on a single trait.

### Reproductive Function, Gamete Viability and Fruit-Set

Fruit yield in vegetables crops is a function of fruit numbers and fruit size. There is a strong and positive correlation between fruit-set and gamete viability (Prasad et al., 2017). Gamete functions (pollen and ovule) is the most important factor for fruit-set under HS. In tomato, fruit-set has been shown to correlate with pollen viability (Firon et al., 2006). In general, heat tolerant genotypes maintain higher pollen viability compared to heat susceptible genotypes (Dane et al., 1991). Gamete functions depend on its viability, which can be evaluated by viability assays like staining, *in-vitro* and *in-vivo* germination of pollen, and ovule function. Genotypes are known to differ in gamete viability under HS stress. Singh et al. (2015) concluded from their research on tomato that traits like fruit-set and pollen viability could be used as a strategy to screen genotypes for HS. In general, the combination of gamete viability and fruit-set provide tolerance to HS (Paupière et al., 2017b; Pham et al., 2020). Similarly observations were also made on peppers (Aloni et al., 2001; Reddy and Kakani, 2007).

Cardinal temperatures (Tmin, Topt, and Tmax) for pollen grain germination can be used to screen germplasm for HT stress tolerance. Results from *in-vitro* studies showed that genotypes varied in response to temperature for cardinal temperatures, and the differences in cardinal temperatures were mainly responsible for tolerance/susceptibility of genotypes to HT stress in soybean (Djanaguiraman et al., 2019) and peanut (Kakani et al., 2002). The genotypes having higher ceiling temperature (Tmax) for pollen germination values tend to be HT tolerant in most cases. Cardinal temperature for pepper were different among susceptible and tolerant cultivars (Reddy and Kakani, 2007) and can be used to identify temperature tolerant or sustainable genotypes of pepper (Gajanayake et al., 2011). All the aforementioned traits based on leaf function are used collectively to select heat tolerant cultivars. Though many studies have successfully employed one trait for selection of heat tolerant genotypes, a combination of these traits reflects a better status of heat tolerance rather than relying on a single trait.

## Omics Approaches

### Genomics

Various modern genome-based technologies can be used to introduce genetic variations for HS tolerance into plants. Under high-temperature stress, plants activate a complex chain of molecular responses, including heat-stress-responsive genes that control primary and secondary metabolism, transcription, translation, and lipid signaling, or protein modifications, including phosphorylation HS transcription factors (HSFs) that regulate differential expression of HSPs (Janni et al., 2020). HSPs and HSFs are key players in the acquisition of the HS response. HSFs are mainly involved in sensing and relaying the HS signal to activate the response (Mittler et al., 2012). Genome-wide associated studies (GWAS) have been conducted on a few vegetable crops to search for novel genes and transcription factors associated with heat tolerance. Genomic studies on cabbage (*Brassica rapa* ssp.) disclosed the role of differentially expressed long non-coding (lncRNAs), mRNAs, and microRNAs. Their expression is associated with phytohormones such as salicylic acid (SA) and brassinosteroids (BRs), possibly involved in heat tolerance. Of these, 25 lncRNAs were co-expressed with ten heat-responsive genes (Wang A. et al., 2019). NAC, a large family of transcription factors, was analyzed in cabbage; 188 genes were identified that play a major role in resistance to high-temperature stress (Ma et al., 2014). Analysis of the potato Hsp 20 gene family revealed 48 putative Hsp20 (StHsp20) that accumulated under heat treatment. Different levels of these transcripts were upregulated during different HS exposures. The transcription of HSPs are regulated by HSFs that play an important role in imparting thermotolerance in plants (Zhao P. et al., 2018). Guo et al. (2015) characterized 35 putative Hsp 20 genes (CaHsp20) located on 12 chromosomes in thermotolerant (R9) and thermosensitive (B6) lines of pepper in four tissues (roots, stem, leaves, and flowers). Under high temperature stress (40°C), most of the CaHsp20 genes had higher expression in both lines, more so in the thermosensitive line. Chidambaranathan et al. (2018) identified 22 Hsfs in the desi (ICC4958) and kabuli (CDC Frontier) genomes of chickpea (15-day-old seedlings; heat treatment of 35 ± 2°C). Field analysis was undertaken to compare the expression pattern at the podding stage. HS at the seedling and pod development stages upregulated the expression of *CarHsfA2, A6a, A6c*, and *B2a*, indicating their role in conferring HS tolerance in chickpea. Yang et al. (2016) recorded 26 HSF (Sly HSF) genes in tomato, with HS (38°C) increasing the expression of most, especially SlyHSF-05/07/13/18/20/23/24. Expression of the SlyHSF-18 gene increased manifold compared to the control, indicating its strong response and correlation to high temperature sensitivity. Moreover, SlyHSF-02 was the main regulator for activating the heat response and acquiring thermotolerance in tomato.

### Transcriptomics

Transcriptomics refers to the study of the transcriptome [entire set of transcripts (mRNA, tRNA, and rRNA, miRNA, siRNA, snRNA, snoRNA, and lncRNA)] expressed in a cell, tissue, organ, or organism. It represents all RNA synthesized, including protein-coding, non-coding, spliced, polyadenylated, and RNA-edited transcripts (Imadi et al., 2015). Transcriptomics reveals the molecular mechanism underlying the phenotype and explains how genes are expressed and interconnected (Jha et al., 2017). High throughput methods (microarray, RNA sequencing, RT-PCR) are used to analyze the expression level of multiple transcripts in different conditions. Several transcriptome studies in vegetable crops under HS have revealed the molecular basis for heat tolerance.

Transcriptome analysis in heat-stressed spinach (42°C for 15 days) revealed the expression of 4,145 transcripts (2,420 upregulated and 1,725 downregulated) in heat-tolerant and heat-sensitive genotypes (Guo et al., 2020). An enrichment analysis showed that the major metabolic difference between tolerant and sensitive genotypes was carbohydrate metabolism (Guo et al., 2020). Similarly, transcriptome analysis revealed 23,000–30,000 expressed genes in soybean seeds and differentially expressed genes (DEGs; 5–44% of expressed genes) (Gillman et al., 2019). The DEGs were measured at high temperature in mature, imbibed, and germinated seeds in a heat-tolerant (PI 587982A) and conventional high-yielding variety (S 99-11986), with 7,789 DEGs common between genotypes, 11,833 common between mature and imbibed seeds, and 13,344 common between imbibed and germinated seedlings (Gillman et al., 2019). In capsicum, seedling transcriptomics revealed 3,799 DEGs in R597 (heat-tolerant genotype) and 4,010 DEGs in S590 (heat-sensitive genotype), related to hormones, HSPs, transcription factors, and calcium and kinase signaling (Li et al., 2015). Further, R597 had higher expression of transcription factors and hormone signaling genes than S590 (Li et al., 2015). Transcriptomic analysis of heat-tolerant PS-1 and heat-sensitive H-24 tomato genotypes under HS (40°C for 1 h) revealed upregulated genes associated with protease inhibitors, HSPs, and transcription factors, manifold higher in the tolerant genotype than the sensitive genotype (Sadder et al., 2014).

### Proteomics

Proteomic analysis in heat-stressed radish leaves (advanced inbred line NAU-08Hr-10) revealed eleven deferentially expressed proteins, of which four belonged to HSPs, four to energy and metabolism, two to redox homeostasis, and one to signal transduction (Zhang et al., 2013). Comparative proteome analysis of heat-tolerant (JG 14) and heat-sensitive (ICC16374) chickpea genotypes under HS during anthesis revealed that 482 heat-responsive proteins (related to photosynthesis, energy metabolism, and signaling molecules) were synthesized in higher amounts in the heat tolerant genotype compared to the sensitive genotype (Parankusam et al., 2017). Proteomics of spinach (50-day-old) exposed to 37/32°C for 24, 48, or 72 h identified heat-stress-responsive proteins in heat-tolerant (Sp75) and heat-sensitive (Sp73) lines (Li et al., 2019). The abundance pattern indicated that HS inhibited photosynthesis, initiated ROS scavenging pathways, and sped up carbohydrate and amino acid metabolism. A comparative proteomic study showed that heat-sensitive genotypes have a lower ability for photosynthetic adaptation, osmotic homeostasis, and antioxidant enzyme activities than heat-tolerant genotypes (Li et al., 2018). Ahsan et al. (2010) used a proteomics approach to study the tissue-specific protein expression pattern in heat-stressed soybean seedlings (40 ± 2°C for 12 h), identifying 61, 54, and 35 differentially expressed proteins in roots, leaves, and stem, respectively. Many of the proteins related to HSPs and the antioxidant system were upregulated.

### Metabolomics

Recent metabolite profiling has focused on important metabolites that govern temperature stress tolerance (Guy et al., 2008). Wang J. et al. (2019) studied the metabolism of heat-tolerant (17CL30) and heat-sensitive (05S180) capsicum cultivars; the tolerant genotype accumulated 94 differentially accumulated metabolites (DEM) while the sensitive genotype accumulated 108 DEM. Both genotypes shared common metabolites, but they were more highly expressed in tolerant genotypes. Metabolite profiling of tomato anthers exposed to 38°C for 2 h revealed that flavonoids (alkaloids and flavonoids in young microspores) protect against HS (Paupière et al., 2017a,b). A metabolomics study on heat-stressed soybean seeds revealed 275 metabolites that comprised antioxidants, including ascorbate precursors, tocopherol, flavonoids, phenylpropanoids, which were more enriched in tolerant than sensitive genotypes (Chebrolu et al., 2016).

## Molecular Breeding

Of late, molecular breeding has emerged as one of the important tools to identify progeny plants possessing the targeted genes/QTLs including the presence of several genes or ascertain the amount of genome of recurrent parent in a plant. Molecular breeding relies on molecular markers and hence the outcome, unlike the phenotyping, is not influenced by environmental factors. The molecular breeding has been exploited successfully in crop breeding and has led to the development of crop varieties possessing resistance to diseases or varieties with resistance genes pyramids (Janni et al., 2020). Molecular breeding methods to improve heat tolerance include (i) transfer of quantitative trait loci, (ii) marker-assisted selection. Other methods include marker assisted recurrent selection, marker-assisted pyramiding, and single nucleotide polymorphism. These methods pave the way for breeding stress tolerance in plants (Collard and Mackill, 2007). These methods pave the way for breeding stress tolerance in plants (Collard and Mackill, 2007).

### Quantitative Trait Loci

QTL is a stretch of genomic regions on a chromosome that is linked to a quantitative trait. Usually, this stretch contains several genes and each QTL contribute partially to the trait in question; and hence, several QTLs together govern a trait. In molecular breeding, whole QTL is transferred to the recurrent parent utilizing markers flanking to the QTLs and sometimes using markers present within the QTL region. The exploitation of molecular breeding for QTLs transfers in breeding programs, a QTL must be well-defined and demonstrated to be linked to a particular trait (Collard and Mackill, 2009). Heat tolerance is a polygenic trait governed by several genes (Golam et al., 2012) and several QTLs. Unprecedented advances in genomics, especially molecular marker development, have identified numerous QTLs contributing to HS tolerance by dissecting various traits ranging from phenological, physiological, biochemical, reproductive biology to yield and yield-related traits (Lucas et al., 2013; Wen et al., 2019; Song et al., 2020; Jha et al., 2021; Vargas et al., 2021) in various vegetable crops, including bottle gourd *(Lagenaria siceraria)*, cowpea (*Vigna unguiculata* [L.] Walp.), common bean, chickpea, chili, and tomato (Table 5). In broccoli (*Brassica oleracea* var. *italica*), five QTLs were identified under HS—QHT_C02, QHT_C03, QHT_C05, and QHT_C07 from the heat-tolerant parent and QHT_C09 from the heat-sensitive parent, with a positive epistatic co-relation between QHT_C03 and QHT_C05 for heat tolerance and APX activity was co-located with QHT_C03 (Branham et al., 2017). Likewise, QTLs such as QHT_C02, QHT_C05, and QHT_C09 were co-located with the AP2 gene governing floral development under HS (Aukerman and Sakai, 2003). Similarly, the meristem identity gene (TFL) was associated with QHT_C02 (Duclos and Björkman, 2008). Subsequently, two novel QTLs contributing to heat tolerance were uncovered by phenotypic evaluation of double haploid-based mapping population for two consecutive summer seasons and by employing QTL-seq approach in broccoli (Branham and Farnham, 2019). Recently, subjecting genome wide association (GWAS) study of one hundred forty two lines unearthed a total of fifty seven significant marker trait associations for various physiological and yield related traits under heat stress in *Brassica rapa* (Chen et al., 2022). In tomato, Xu et al. (2017) mapped 13 QTLs for heat tolerance linked with reproductive traits, including pollen viability, pollen number, style protrusion, anther length, style length, flower per inflorescence, and inflorescence number. These QTLs showed additive effects and no epistatic interaction. Likewise, six QTLs linked to fruit set in tomato at high temperatures were identified (Grilli et al., 2007). Based on evaluating recombinant inbred lines and introgression lines developed from *Solanum lycopersicum* var. “MoneyMaker” × *S. pimpinellifolium* across multi environments under high temperature stress enabled in identification of 22 QTLs related to reproductive traits (flower number fruit number and fruit set proportion) on LG1, 2, 4, 6, 7, 10, and 11 explaining phenotypic variation from 4 to 13% (Gonzalo et al., 2020). In combination of phenotypic assessment of leaf cell membrane stability by applying heat stress in F_2_ derived mapping population with QTL-seq approach in F_2_ derived mapping population assisted in uncovering a total of seven QTLs *qHT1*. *1, qHT2*. *1, qHT2*. *2, qHT5*. *1, qHT6*. *1, qHT7*. *1*, and *qHT8*. *1* conferring heat tolerance in bottle gourd (Song et al., 2020). Likewise, employing conventional QTL mapping and QTL-seq analysis allowed in identifying a total of five major QTLs *qHII-1-1, qHII-1-2, qHII-1-3, qHII-2-1*, and *qCC-1-5* (*qREC-1-3*) related to heat injury index under heat stress in tomato (Wen et al., 2019). The authors performed the functional validation of the underlying selected four potential candidate genes *SlCathB2, SlGST, SlUBC5*, and *SlARG1*. To decipher genetic basis of heat tolerance in cucumber, QTL analysis of mapping population developed from “99281” (heat-tolerant) × “931” (heat-sensitive) population phenotypically evaluated during summer 2018, 2019, and 2020 allowed to identify one major QTL *qHT1.1* on LG1 (Liu et al., 2021). There were 98 genes underlying this QTL. Of these identified genes, expression of *Csa1G004990* candidate gene was higher in “99281” than “931” genotype rendering it heat tolerant. In order to shed light into the functional role of HSP20 contributing to heat tolerance, in *Cucurbita moschata*, genome wide bioinformatic analysis enabled in unveiling 33 *HSP20* genes across the genome (Hu et al., 2021). Functional validation of CmoHSP20-7, 13, 18, 22, 26 and 32 genes indicated their possible role in heat tolerance in *Cucurbita moschata* (Hu et al., 2021).

**Table 5:** List of selected QTLs contributing to heat tolerance in vegetable crops.

**Crop**	**Mapping population**	**Trait used**	**Name of gene/** **QTL**	**Type of** **marker**	**Linkage groups**	**Phenotypic** **variation**	**References**
**Bottle gourd** (*Lagenaria siceraria*)	L1 × L6	Relative electrical conductivity	*qHT1.1, qHT2.1, qHT2.2, qHT5.1, qHT6.1, qHT7.1, and qHT8.1*	SNP	1, 2, 5, 6, 7, 8	–	Song et al., 2020
**Cowpea** (*Vigna unguiculata*)	CB27 x IT82E-18, RIL 141	–	*Cht−1, Cht−2, Cht−3, Cht−4, Cht−5*	SNP	2, 3, 6, 7, 10	11–18%	Lucas et al., 2013
	IT93K-503-1 x CB46, RIL 113; IT84S-2246 x TVu146, RIL 136	Seed coat browning	*Hbs-1, Hbs-2 and Hbs-3*	SNP	1, 3, 8	6–77%	Pottorff et al., 2014
**Common bean** (*Phaseolus vulgaris*)	IJR × AFR298, RIL	Reproductive trait and yield and yield traits	32 QTLs	SNP	1, 2, 3, 4, 5, 8, 9, 10	7.8–36%	Vargas et al., 2021
**Chickpea** (*Cicer arietinum*)	DCP 92-3 × ICCV92944 RIL(184)	Phenological, physiological and yield related traits	77 QTLs	SNP	LG1–LG8	5.9–43.5%	Jha et al., 2021
	DCP 92-3 × ICCV92944F2(206)	Phenological and physiological traits	2 QTLs	SSR	–		Jha et al., 2019
	ICC 4567 × ICC 15614, RILs(292)	Yield and yield traits	4 QTLs	SNP	CaLG05, CaLG06	–	Paul et al., 2018
	GPF2 × ILWC292, RIL	Phenological, physiological and yield related traits	28 + 23 QTLs	SNP	All LG groups except LG8	5.7–13.7%	Kushwah et al., 2021
**Chili** (*Capsicum annuum*)	AVPP0702 × Kulai, backcross	Reproductive and yield trait	Hsp70 and sHsp gene	SSR	–	–	Usman et al., 2018
**Tomato** (*Lycopersicon esculentum*)	Nagcarlang × NCHS-1180 F2	Reproductive traits; viz., pollen viability, pollen number, style length, anther length; inflorescence number and flowers per inflorescence	*qPV11, qPN7, qSP1, qSP3, qAL1, qAL2, qAL7, qSL1, qSL2, qSL3, qFPI1 qIN1, qIN8*	SNP	1, 2, 3,7, 8, 11	10.5–38.7%	Xu et al., 2017
	MAGIC population	Yield components, phenology andfruit quality	69 plasticity QTLs	SNP			Bineau et al., 2021
	LA1698 × LA2093	Relative electrical conductivity REC), chlorophyll content (CC) and maximum photochemical quantum	5 major QTLs *qHII-1-1, qHII-1-2, qHII-1-3, qHII-2-1*and *qCC-1-5 (qREC-1-3)*	SNP	1, 2	16.48%	Wen et al., 2019
	*Solanum lycopersicum* var. “MoneyMaker” × *S. pimpinellifolium* accession TO-937RIL and IL	Reproductive traits viz., flower number, fruit number per truss and percentage of fruit set, stigma exsertion (SE), pollen viability (PV), tip burn	22 QTLs	SNP8K SNP SOLCAP Infinium chip	1, 2, 4, 6, 12	3.6–12.8%	Gonzalo et al., 2020

In cowpea, five QTLs governing pod set at high temperature, namely Cht-1, Cht-2, Cht-3, Cht-4, and Cht-5, with CB 27 line of cowpea donating alleles for four QTLs (Cht-1, Cht-2, Cht-3, Cht-4) and IT82E-18 contributing alleles for Cht-5 (Lucas et al., 2013). Combinations of any of the four QTLs with Cht-5 positively correlated with heat tolerance in cowpea. Further, the presence of all five QTLs in the same line had the strongest positive correlation with heat tolerance (Lucas et al., 2013). Recently, four QTLs were identified in chickpea that conferred heat tolerance for filled pods (qfpod03_6), grain yield (qgy03_6), total seed number (qvs05_6), and pod set (q% podset08_6) using recombinant inbred lines produced from ICC 4567 (heat-sensitive) × ICC 15614 (heat-tolerant) lines (Paul et al., 2018). One QTL (qTBP5.2) was detected in lettuce, governing the tip-burn resistance trait, therefore beneficial in breeding programs (Jenni et al., 2013). The information on genomes of crops is expanding rapidly. The sequencing coupled with resequencing will generate more information that will subsequently be used to gather detailed knowledge of QTLs and genomic bases of heat tolerance in crops. The closely-related crops share syntenic relationships and possess similar genomic regions with each other. In the forthcoming years, comparative genomic analysis and advancements in knowledge of molecular biology might allow us to transfer heat tolerant regions from one crop to another, thereby expanding the repository of cold tolerance in crop plants.

## Marker-Assisted Selection

As mentioned earlier, phenotype-based selection is prone to environmental conditions sometimes leading to erroneous conclusions especially if trait is complex and conferred by polygenes or QTLs. Under such circumstances, genotype-based selection is more effective, precise and fast as compared to phenotypic selection. Genotype-based selection rather than phenotype-based selection is possible using markers linked to gene of interest. Genotype-based selection utilizes DNA markers that are linked tightly to the gene(s) of interest (Collard and Mackill, 2007). For MAS, first step is to identify markers linked to the gene or QTL using either mapping populations or association mapping where a panel of genotypes is used to identify liked markers. Subsequently, these markers are used to ascertain transfer of the gene to the progeny populations. Different types of markers, such as RFLP (restricted fragment length polymorphism), AFLP (amplified fragment length polymorphism), SSR (single sequence repeat), and SNPs (single nucleotide polymorphisms), can be detected, and the amount of variation in each marker can be determined. Using this approach, gene mapping and identifying gene associations with particular traits are useful for genetic crop improvement (Ruane and Sonnino, 2007).

Paul et al. (2018) identified SNP markers linked to QTLs for heat tolerance traits (50% flowering, podding behavior, total filled pods, % pod set, total seed number, grain yield, biomass, harvest index, 100-seed weight) in chickpea RILs (heat-tolerant ICC 15614 × heat-sensitive ICC 4567). Composite interval mapping analysis affirmed two genomic regions (CaLG05 and CaLG06) with four QTLs (grain yield, total seed number, total filled pods, % pod set). A GWAS used 16,877 SNPs to identify marker-trait associations (MTA) in 135 diverse pea lines exposed to >28°C in the field to understand the genetic basis for heat tolerance (Gali et al., 2019). The study identified 32 MTAs and 48 candidate genes associated with various traits, including chlorophyll concentration, photochemical reflectance index, canopy temperature, reproductive stem length, internode length, pod number, with the potential for developing heat-tolerant cultivars (Tafesse et al., 2020). Lin et al. (2006) identified 14 RAPD markers linked to heat tolerance traits (flower number, fruit number, fruit set, yield) in tomato RILs derived from CL5915 (heat-tolerant) and L4422 (heat-sensitive) under HS. Developing heat tolerant *Capsicum annuum* through transferring heat shock protein encoding gene *Hsp70* and *sHsp* from AVPP0702 into Kulai an elite *C. annuum* cultivar by adopting marker assisted back crossing approach is notable illustration of marker assisted breeding for heat tolerance (Usman et al., 2018). Likewise, three non-synonymous SNPs identified in the *qHT2.1* major effect QTL in bottle gourd (Song et al., 2020) and non-synonymous SNP identified in the *QHT_C09.2* QTL regions in broccoli (Branham and Farnham, 2019) contributing to heat tolerance, which could be potentially used as candidate markers for screening heat tolerant bottle gourd and broccoli genotypes.

## Transgenics

Altering the genetic makeup of vegetable crops is a possible solution for developing crops that can grow and reproduce well under increasing temperatures. Plants have an inherent ability to endure supra optimal temperatures (“basal thermotolerance” or “acquired tolerance to increasing temperature”) (Grover et al., 2013). The level of thermotolerance varies between plant species depending on their genetic makeup and specific expression of defense-related genes, however, levels of thermotolerance vary in different plant species again due to differences in genetic makeup of the plant species. Even within a species, genotypes differ for reaction (tolerance or sensitive) to HS owing to varying genetic makeup. Considerable number of genes/QTLs conferring tolerance to HS has been identified in vegetable crops and these genes/QTLs can be transferred from heat-tolerant genotypes to heat-sensitive genotypes using transgenic approaches to develop genetically modified heat tolerant crops. Genes expressed in heat-tolerant crops can be transferred to heat-sensitive crops using transgenic approaches to develop genetically modified heat-tolerant crops. Candidate genes for development of transgenics for heat tolerance are HSP, compatible osmolyte, and antioxidant levels, and detoxifying pathways (Parmar et al., 2017).

### Manipulating HSPs

Many vegetable crops have been manipulated for increased expression of HSPs. For instance, in tomato, overexpression of trehalose-6-phosphate synthase/phosphatase (TPSP) gene derived from *Escherichia coli* increased the expression of HsfA1, HsfA2, and HsfB1, which was linked to escalating Hsp17.8, ER-sHsp and Mt-sHsp levels to impart heat tolerance (Lyu et al., 2018). Similarly, overexpression of small heat shock protein (CaHsp 25.9) improved thermotolerance in Capsicum transgenic lines (R9 and B6) under HS, decreasing MDA content and increasing proline and SOD content (Feng et al., 2019). In transgenic potato lines, overexpression of the A2 HSc70 (Heat-Shock Cognate) allele-maintained tuber yield at elevated temperature (Trapero-Mozos et al., 2018).

### Manipulating Antioxidants

HS causes oxidative damage in plants; therefore, developing transgenics with enhanced antioxidative mechanisms may enhance thermotolerance in plants. Antioxidant mechanisms were manipulated in pea by incorporating heat shock factor gene (HsfA1d) from *Arabidopsis thaliana*. Under HS (42°C), transgenic pea plants had five-fold higher expression of HsfA1d than wild pea, decreasing H_2_O_2_ accumulation, and higher SOD and APX activities and proline content (Shah et al., 2020). Tang et al. (2006) developed transgenic potato plants (SSA plants) expressing Cu/Zn SOD and APX gene in chloroplasts under the control of a SWPA2. The transgenic plants had less damage induced by methyl viologen than non-transgenic plants. In the same study, photosynthetic activity decreased by 29% in non-transgenic plants but only 6% in transgenic plants under HS (42°C for 20 h). Overexpression of cytosolic APX (cAPX) in transgenic tomato (*Lycopersicon esculentum* cv. Zhongshu No. 5) under HS (40°C for 13 h) resulted in several-fold higher APX activity than wild plants, reducing electrolyte leakage (24% in A9 line and 52% in A16 line) compared with wild plants. Similarly, overexpression of cAPX in transgenic tomato increased tolerance HS (Wang et al., 2006).

### Cross-Talk Between HSP and Redox Mechanism

**Figure 4:**
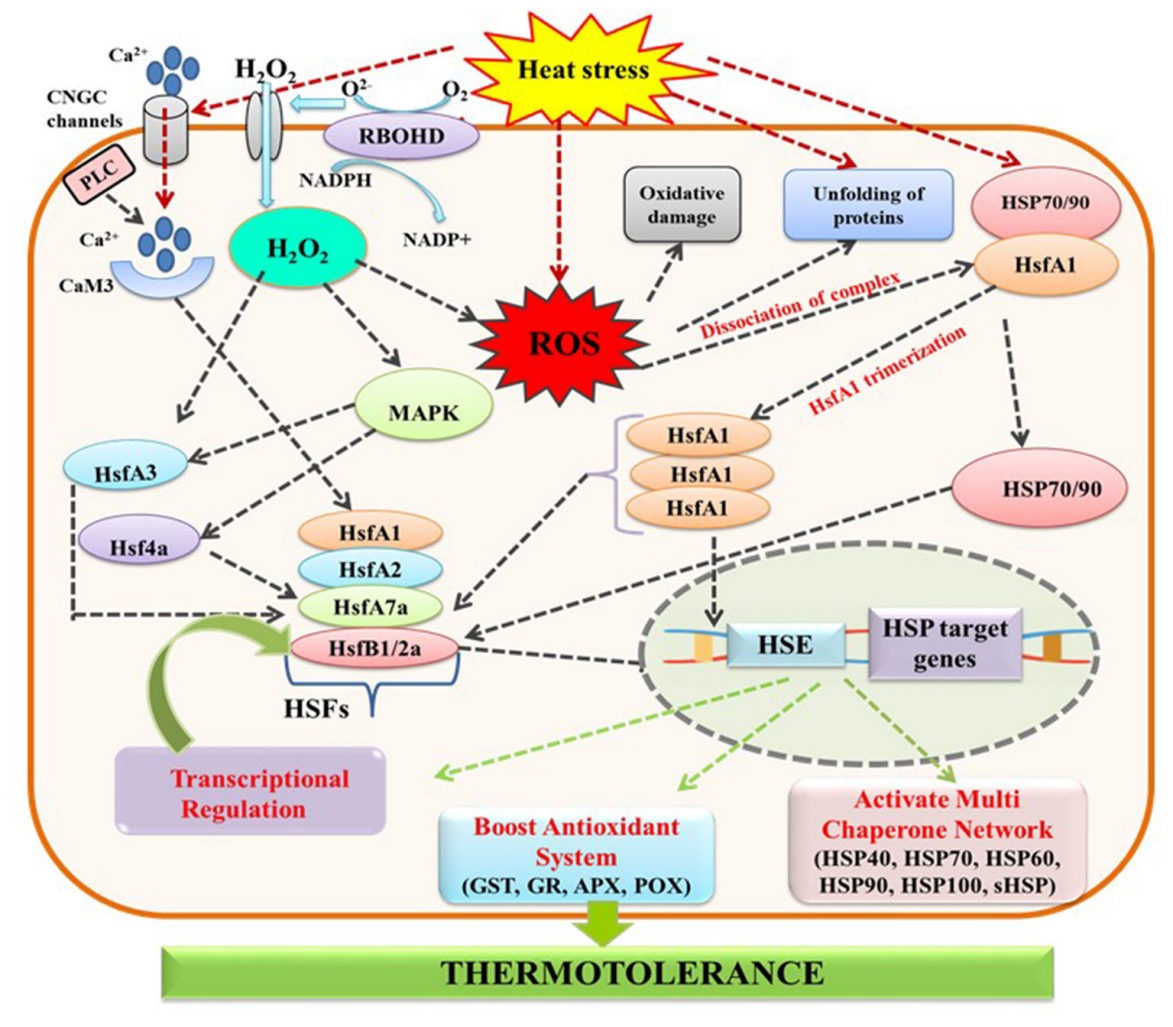
Cross talk between HSPs and redox reaction: **-**Heat stress imposes damages to plant like increased membrane fluidity, unfolding of proteins, ROS production and dissociation of HSP70/90-HsfA1 complex. To endure HS, Plants activate various mechanisms to preserve their adaptation. First such mechanism is the activation of cyclic nucleotide gated calcium (CNGC) channels that result in the movement of Ca^2+^ions in to cytoplasm and bind with Calmodulin Protein (CaM3) forming the Ca^2+^-CaM3 complex and help in the activation of Heat shock factors (HSFs). Second mechanism involves Phosphoinositol signaling pathway that also lead to the influx of more Ca^2+^in to the cytoplasm and merge with Ca^2+^-CaM3 pathway. Another mechanism during HS is the activation of ROS signaling network by Respiratory Burst Oxidase Homolog D (RBOHD) that produce O^2−^ which is converted in to H_2_O_2_ that is involved in the induction of HSFs activation. ROS like H_2_O_2_ also activate the HSFs complex through mitogen activated protein kinase (MAPK). On activation, HSFs move to the nucleus and activate HSE and HSP target genes. HS also lead to the dissociation, of HSP70/90-HsfA1 complex; on dissociation HsfA1 undergoes trimerization that further activates the HSFs complex in the cytosol and Heat shock element (HSE) in the nucleus. Their activation has many positive effects on the cellular metabolism like transcriptional regulation, activation of antioxidant system and multi chaperone network (HSP60, HSP70, HSP90, HSP100, and sHSP) that may lower down the ROS levels in the cell and help in achieving thermotolerance.

Equilibrium between ROS generation and ROS scavenging is disturbed by the high temperature stress (Foyer and Noctor, 2005). One of the best strategies adopted by the plant cells is the production of HSPs on exposure to high temperature (Wang et al., 2004). HSPs positively affect thermotolerance by protecting ROS scavenging system and actively resulting in lower ROS concentration. HSPs also enable protein refolding, preventing aggregation of non-native proteins and stabilize polypeptides and membrane under stress conditions (Scarpeci et al., 2008). It is unclear whether there is specific interaction between HSPs and ROS scavenging machinery but ROS accumulation is reduced *via* HSP induced ROS scavenging activity. Hence the cross-talk between production of HSFs/HSPs and ROS scavenging activity play important role in acclimation (Kang et al., 2022). The communication between ROS and HSFs involve Mitogen Activated Protein Kinase (MAPK). ROS dependent phosphorylation can play vital role in HSF activation (Driedonks et al., 2015). MAPK3 and MAPK6 are the key players which are activated by H_2_O_2_ and further phosphorylate the HSFs, for instance in tomato, heat induced MAPK transduces the heat stress signal *via* HSFA3 (Link et al., 2002). Induction of heat shock transcription factors HsfA2 and HsfA4 is reported to be regulators of genes associated with ROS mitigation. HsfA4A is the principle candidate to function as H_2_O_2_ sensor (Scarpeci et al., 2008). At transcriptional level, HSPs are regulated by HSFs that bind to the conserved regulatory element of heat shock element (HSEs) and act as promoter for Hsp genes. Under stress conditions ROS mainly H_2_O_2_ functions as signal transduction molecule and cause HSF activation. ROS enhances the dissociation of HSP and HSF complex and promote the HSF trimerization and relocate the same to the nucleus leading to activation of the expression of HSPs and other heat responsive genes (Ul Haq et al., 2019) (Figure 4).

## Agronomic Approaches

By employing improved agronomic practices for different crops has improved crop yields. These practices include better soil, water, nutrient, weed, and pest management strategies, selection of varieties, and appropriate planting times and planting densities, and more and more (HanumanthaRao et al., 2016). Agronomic practices control soil temperature by minimizing the evaporation (Ferrante and Mariani, 2018) helping the cultivators with sustained water use, proper fertilizer use, and improved land maintenance, consequently improving crop quality and quantity. In addition, agronomic practice also helps with increased soil physical, chemical and microbial status. These help with water and nutrient availability and plant uptake. Agronomic practices for increasing vegetable crop yields that are efficient, cost-effective, and easily adaptable for HS management are described below.

Land preparation for planting involves tillage, seedbed shaping, and mulching. These practices depend on the soil type, physical and chemical properties. Sandy loam soils are best for raising vegetables such as potato, cauliflower, lettuce, cabbage, and tomato. Tillage includes breaking up/loosening the soil by plow, favoring seed germination, and proper seedling growth. Tillage also helps control weeds, aerate soil, and bury the previous crop's residues; the tillage method varies between crops (Kladivko, 2001). However, the same benefits can be obtained with no-till or minimum tillage practices that minimizes soil disturbance and helps with building of soil organic carbon over time. Mulching is a process of covering the soil with chopped residues; it has many benefits, including reduced soil erosion and water loss, which maintain soil temperature (Mulumba and Lal, 2008). Use of conservation agricultural practices with minimum soil disturbance, grass mulch cover and crop rotations not only significantly increased yield of green pepper but also decreased irrigation water use and runoff, while increasing percolated water in the root zone (Belay et al., 2020). Similarly, improved yields of tomato, cucumber and bitter guard were observed under conservation agriculture (Paudel et al., 2020). Conservation agricultural practices in vegetable production systems has shown to increase soil organic matter and nutrients (Belay et al., 2022). Irrigation increases soil moisture, decreasing soil temperature (by 2°) compared to non-irrigated soil (Lobell and Bonfils, 2008). Water quality and supply varies according to soil type, crop (warm- or cool-season), and weather conditions. Generally, vegetable crops are irrigated at 4–6-day intervals during summer and 14–15-day intervals during winter to reduce the high-temperature effects. Many modern technologies for irrigation are available that minimize water use, such as drip or trickle irrigation and overhead micro-sprinklers.

Variety selection is a successful agronomic approach for achieving high yields under high-temperature stress. Selection characteristics include high yield, disease resistance, maturity group, and grain quality (Pedersen, 2003). Suitable crop genotypes need to be early maturing and high yielding to escape heat by completing their life cycle early and thus perform better under HS (Sekhon et al., 2010). Furthermore, shifting the sowing time (early or late) is another strategy to avoid HS and avoid heat induced yield reduction as has been reported in mungbean (up to 50%) and soybean where yield declined tremendously by delay in the sowing date (Coventry et al., 1993; Miah et al., 2009). The goal of selection of crop duration and time of planting is to avoid HS during sensitive stages of reproductive development. In contrast, late sowing has been used to screen large populations of chickpea (Gaur et al., 2013), mungbean (Sharma et al., 2016), and lentil (Sita et al., 2017) genotypes for heat tolerance, some of which have been released (e.g., chickpea ICCV 92944) (Gaur et al., 2013). Heat-tolerant varieties of some vegetable crops are listed in Table 6. Hence, determining the ideal sowing time and selection of heat tolerant varieties is crucial for growth, development, and yield of crops.

**Table 6:** Heat-tolerant varieties of some vegetable crops.

**Crop**	**Trait indicating tolerance**	**Heat-tolerant varieties**	**References**
**Broad bean** (*Vicia faba*)	Seed yield	C.52/1/1/1	Abdelmula and Abuanja, 2007
**Broccoli** (*Brassica oleracea* var. *italica*)		Gypsy and Packman	Farnham and Bjorkman, 2011
**Cabbage** (*Brassica oleracea* var. *capitata*)	Cell membrane thermostability	Sousyu	Chauhan and Senboku, 1996
		ASVEG#1	Fu et al., 1993
**Capsicum** (*Capsicum annuum*)		Mr. Lee No. 3 selex, CCA-119A, Susan's Joy, CCA-3288	Dahal et al., 2006
		IIHR Sel.-3	Devi et al., 2017
**Cauliflower** (*Brassica oleracea* var. *botrytis*)		IIHR316-1, IIHR371-1 and PusaMeghna	Devi et al., 2017
**Chickpea** (*Cicer arietinum*)		ICCV07110, ICCV92944	Kumar et al., 2013
**Common bean** (*Phaseolus vulgaris*)	Chlorophyll fluorescence	Ranit and Nerine RS	Petkova et al., 2007
		IIHR-19-1	Muralidharan et al., 2016
**Cowpea** (*Vigna unguiculata*)		IT93K-452-1, IT98K-1111-1, IT93K-693-2, IT97K-472-12, IT97K-472-25, IT97K819-43 and IT97K-499-38.	Timko and Singh, 2008
**Lettuce** (*Lactuca sativa*)		S24 and S39	Han et al., 2013
**Mungbean** (*Vigna radiata*)	Seed yield	NFM-6-5 and NFM-12-14	Khattak et al., 2006
	Biomass, number of flowers, pods and seeds weight/plant	EC693357, EC693358, EC693369, Harsha and ML1299	Sharma et al., 2016
**Okra** (*Abelmoschus esculentus*)	Yield (fruit number)	L2-11 and L4-48	Hayamanesh, 2018
**Potato** (*Solanum tuberosum*)	Tuber yield and dry matter	HT/92-621 and HT/92-802	Minhas et al., 2001
**Pea** (*Pisum sativum*)		IIHR-1 and IIHR-8	Muralidharan et al., 2016
**Soybean** (*Glycine max*)	Pollen traits	45A-46	Alsajri et al., 2019
	Pollen traits	DG 5630RR	Salem et al., 2007
**Spinach** (*Spinacia oleracea*)	Seed germination	Ozarka II, Donkey, Marabu, and Raccoon	Chitwood et al., 2016
**Tomato** (*Lycopersicon esculentum*)		CL1131-0-043-0-6, CL6058-0-3-10-2-2-2 PusaSadabahar, PusaSheetal, Pusa Hybrid-1	Abdul-Baki, 1991* Devi et al., 2017

### Nutrients/Thermo-Protectants

HS can be alleviated by exogenous application of nutrients or thermo-protectants as a seed pretreatment, foliar spray, or by fertilizer application *via* broadcasting, pellet placement, or band placement (Waraich et al., 2012; HanumanthaRao et al., 2016). Macro-nutrients such as N, P, K, Ca, and Mg are required by plants (>10 mM) and help maintain structural and functional integrity (Waraich et al., 2011). Nutrient deficiencies alter the levels of tolerance to abiotic stresses. During HS, N deficient plants were associated with increased lipid peroxidation, while N supplemented plants tolerated photo-oxidative damage (Kato et al., 2003). Likewise, K deficient plants had reduced translocation of photo-assimilates to the sink organ, whereas K application improved the translocation and utilization of photo-assimilates, maintained cell turgidity, and upregulated enzymatic activity under HS (Mengel et al., 2001; Cakmak, 2005), increasing yield by 1.9-fold in Capsicum and 2.4-fold in tomato (Waraich et al., 2012). Similarly, exogenous application of calcium (2 L/ha) increased lettuce production under HS (Almeida et al., 2016).

Micronutrients such as B and Mn also provide heat tolerance of plants by increasing antioxidant activity and alleviating the damage induced by HS stress (Waraich et al., 2011). Other elements such as Se increased enzymatic activity and decreased membrane damage and ROS production in soybean (Djanaguiraman et al., 2005). Seed pretreatment and foliar application of thermoprotectant molecules such as proline, glycinebetaine, salicylic acid, spermidine, putrescine, GABA, ascorbic acid provides thermotolerance to crop plants (HanumanthaRao et al., 2016). For instance, exogenous application of proline mitigated HS effects in chickpea (Kaushal et al., 2011). Ascorbic acid application to mungbean seedlings under HS in a controlled environment improved seedling growth (Kumar et al., 2011). In cucumber, a 1 mM SA foliar spray provided heat tolerance by increasing CAT activity and thus reducing membrane damage and H_2_O_2_ levels (Shi et al., 2006). Similarly, Kaur et al. (2009) reported that exogenous application of SA (10 and 20 μM) to heat-stressed brassica seedlings (40–55°C) improved CAT and POX activities. Pretreatment of SA to mungbean seedlings decreased lipid peroxidation and enhanced antioxidant activity, improving membrane stability (Saleh et al., 2007). In chickpea, a 100 μM SA foliar spray to heat-stressed seedlings (46°C) increased proline content (Chakraborty and Tongden, 2005). Thus, exogenous SA application mitigates the harmful impacts of heat-induced damage by strengthening antioxidative pathways. Foliar spray of Se (8 μM) to cucumber plants exposed to 40/30°C during flower initiation (35–75 DAS) decreased oxidative damage by stabilizing the antioxidative mechanism and increasing ROS scavenging (Balal et al., 2016).

### Microorganisms Imparting Thermotolerance

In addition to other factors, plant-associated microorganisms, including plant-growth-promoting rhizobacteria, endophytic bacteria, and symbiotic fungi, play a significant role in imparting thermotolerance in plants (Grover et al., 2011). Many agriculturally important microbes have been discovered that colonize and promote plant growth and aid in nutrient and disease control through various direct and indirect methods (Singh et al., 2016). The interaction between microorganisms and host plants imparting stress tolerance is a complex process and polygenic in nature. Ali et al. (2009) discovered a thermotolerant strain of *Pseudomonas* sp. AMK-P6 in sorghum that elicits HSPs synthesis under high-temperature stress, and improves biochemical activities by inducing the synthesis of osmolytes such as proline, sugars, amino acids, and chlorophyll. *Pseudomonas putida* NBRI0987, a thermotolerant strain (<40°C) was isolated from the chickpea rhizosphere (Srivastava et al., 2008). A recent study on different rhizobacterial strains of pigeon pea at high temperature (30, 40, 50°C) showed that S1p1 and S12p6 were the most promising strains for plant growth and development, stimulating auxin production, flavonoid production, and siderophore formation (Modi and Khanna, 2018). It would be worth evaluating the effectiveness of these microbes in vegetable crops for induction of thermotolerance.

### Protected Cultivation

Growing vegetables in protected environments on small-scale farms using modern technologies has gained considerable attention for their high yields and quality and regular vegetable supply in the off-season (Sabir and Singh, 2013). Protected cultivation involves manipulating environmental factors such as temperature, humidity, light, water, and soil by designing suitable structures and following appropriate practices (Wittwer and Castilla, 1995). The main practices for protected cultivation are row tunnels, polytunnels, and mulching, which are more beneficial than open-field cultivation with less demand for fertilizers, pesticides, and water (Choudhary et al., 2013). In tomato, using a fogging system for 20 min/h (between 10 a.m. and 4 p.m.) in a hot shade house (>37°C) obtained high fruit yields with fewer physiological disorders (Ro et al., 2021). A similar fogging system improved the antioxidant defense responses in tomato plants (Leyva et al., 2013). Related approaches have been used to cultivate cucumber, capsicum, and lettuce with high yields (Sabir and Singh, 2013).

## Conclusions

Vegetables are a distinct collection of plant-based foods that vary in nutritional diversity and form an important part of healthy diets. They also have great potential for boosting human health. Exposure to high temperatures or HS can directly or indirectly influence the production and quality of fresh vegetables. Several heat-induced morphological damages, such as poor vegetative growth, leaf tip burning, rib discoloration in leafy vegetables, sun burned fruits, decreased fruit size; pod abortion, and unfilled pods are common, which can render vegetable cultivation unprofitable. Key physiological and biochemical effects associated with crop failure include membrane damage, photosynthetic inhibition, oxidative stress, and reproductive tissue damage. Reproductive stage has extensively been studied and found to be more sensitive to HS as it directly affects yields by reducing processes like pollen germination, pollen load, pollen tube growth, stigma receptivity, ovule fertility, and seed filling, resulting in poorer yields. Hence, sound and robust adaptation strategies are needed to mitigate the adverse impacts of HS to ensure the productivity and quality of vegetable crops.

**Figure 5:**
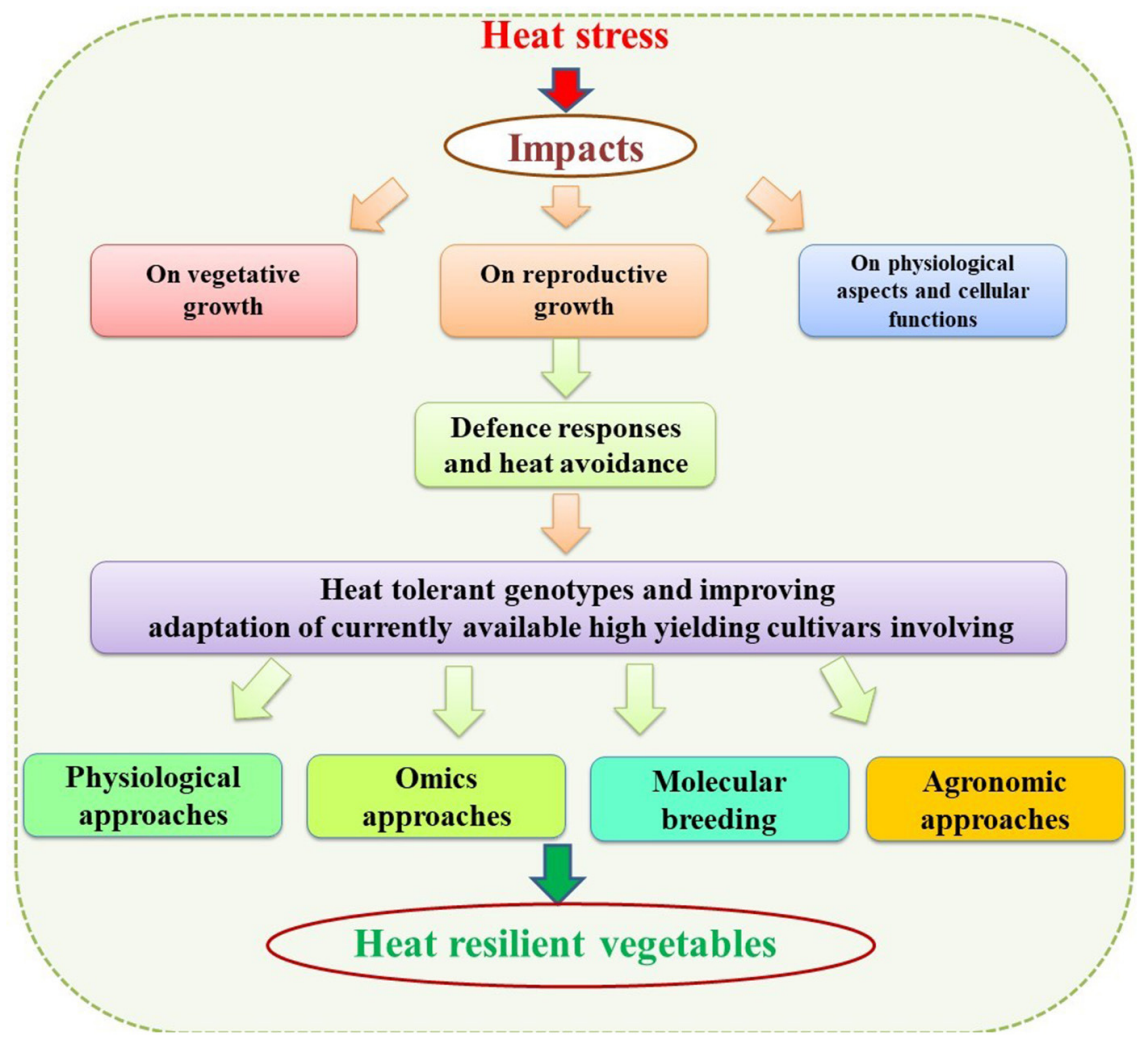
Heat stress has various negative impacts on the plant like reducing vegetative and reproductive growth, interfering with the physiological and cellular functions. To combat such impacts, plant activates multiple responses and heat avoidance mechanisms which can be used to identify heat resilient vegetable crops. Different approaches categorized in this article for this purpose are physiological based, omics based, molecular breeding based and agronomic based. Such possible options will pave the way for improving adaptation and mitigation of heat stress in vegetable crops.

Most important strategy to manage HS is deployment of heat tolerant cultivars (Figure 5). Physiological traits, such as stay-green trait, canopy temperature depression, cell membrane thermostability, chlorophyll fluorescence, relative water content, and stomatal conductance, are especially important in developing high-yielding heat-tolerant varieties/cultivars. Molecular approaches like omics, molecular breeding and transgenics have the potential to enhancing heat tolerance either by transferring heat tolerant genes/QTLs to elite cultivars with the help of molecular markers or elucidating mechanisms of tolerance leading to identification of heat tolerance genes and transferring those across genera or families *via* genetic modifications. Besides these approaches, simple agronomic methods are also important for mitigating HS effects at the grassroots level. Therefore, developing heat-tolerant plant types using physiological, molecular, and breeding-based techniques is essential for sustaining vegetable production systems and human health. Further, these approaches will offer insight into the physiological and molecular mechanisms that govern thermotolerance and pave the way for engineering ‘designer' vegetable crops for better health and nutritional security.

## Data Availability Statement

The original contributions presented in the study are included in the article/supplementary material, further inquiries can be directed to the corresponding authors.

## Author Contributions

All authors listed have made a substantial, direct, and intellectual contribution to the work and approved it for publication.

## Conflict of Interest

The authors declare that the research was conducted in the absence of any commercial or financial relationships that could be construed as a potential conflict of interest.

## Publisher's Note

All claims expressed in this article are solely those of the authors and do not necessarily represent those of their affiliated organizations, or those of the publisher, the editors and the reviewers. Any product that may be evaluated in this article, or claim that may be made by its manufacturer, is not guaranteed or endorsed by the publisher.
